# Multifunctional Valorisation of Pistachio (*Pistacia* spp.) By-Products: A Review of Sustainable Applications in Environmental and Industrial Contexts

**DOI:** 10.3390/ijms27104306

**Published:** 2026-05-12

**Authors:** Tomasz Kowalczyk, Maciej Kowalski, Adam Majchrzak, Laurent Picot, Lucyna Herczyńska, Wirginia Kukula-Koch, Noureddine El Aouad, Przemysław Sitarek

**Affiliations:** 1 Department of Molecular Biotechnology and Genetics, Faculty of Biology and Environmental Protection, University of Lodz, Banacha 12/16, 90-237 Lodz, Poland; 2 Students Research Group, Department of Medical Biology, Medical University of Lodz, 90-151 Lodz, Poland; maciej.kowalski1@student.umed.lodz.pl; 3 Students Research Group, Textile Institute, Faculty of Material Technologies and Textile Design, Lodz University of Technology, Żeromskiego 116, 90-924 Lodz, Poland; 250241@edu.p.lodz.pl; 4 Littoral Environnement et Sociétés UMRi CNRS 7266 LIENSs, La Rochelle Université, 17042 La Rochelle, France; laurent.picot@univ-lr.fr; 5 Textile Institute, Faculty of Material Technologies and Textile Design, Lodz University of Technology, Żeromskiego 116, 90-924 Lodz, Poland; lucyna.herczynska@p.lodz.pl; 6 Department of Pharmacognosy with Medicinal Plants Garden, Medical University of Lublin, Chodzki 1, 20-093 Lublin, Poland; virginia.kukula@gmail.com; 7 Research Team on Natural Products Chemistry and Smart Technologies (NPC-ST), Polydisciplinary Faculty of Larache, University Abdelmalek Essaadi, Tetouan 93000, Morocco; n.elaouad@uae.ac.ma; 8 Department of Medical Biology, Medical University of Lodz, Muszyńskiego 1, 90-151 Lodz, Poland

**Keywords:** bioactive compounds, circular economy, composite development, environmental applications, green nanomaterials, nanotechnology, pistachio by-products, *Pistacia* spp., sustainable materials, waste valorization

## Abstract

The growth of pistachio (*Pistacia* spp.) production, particularly in America, Iran, and Turkey, has resulted in the production of substantial quantities of by-products, including shells, fruit husks, leaves, resins, and tree pruning waste. Although these fractions contain a range of beneficial secondary compounds, they are rarely used. Recent advances in nanotechnology and materials engineering suggest that they can be used as inexpensive, renewable raw materials in the fields of agriculture, the food industry, cosmetics, pharmaceuticals, and environmental engineering. Pistachio by-products have been shown to exhibit antioxidant, antibacterial, antifungal, and anticorrosive properties, making them good candidates for use in active packaging systems and nanoencapsulation. They may also be valuable as fertilisers, animal feed, and composite materials. They have been found to have potential roles in industrial pollutant removal and energy storage, and as ‘green’ precursors for nanomaterials. This review summarises the current knowledge on the multifunctional applications of *Pistacia* spp. by-products, emphasising their importance in implementing circular economic strategies and sustainable industrial development. This study also addresses the practical application of pistachio by-products, as well as the widely appreciated nuts, within the domain of patents, thereby illustrating the prospective pathway of currently emerging solutions in the context of sustainable development.

## 1. Introduction

The progressive degradation of the environment driven by pollution, soil depletion, loss of biodiversity, and unsustainable use of natural resources is a matter of global concern and influences food security, sustainable industrial development, and public health. The 2003 UN High-Level Panel on Threats, Challenges and Change indicated environmental degradation as both a considerable threat to global stability and a biological threat [1,2]. In response, the UN 2030 Agenda for Sustainable Development has prioritised the use of eco-friendly manufacturing methods and circular economy [3]. Hence there is a need to reuse waste from farming and food production as this can improve resource use and reduce damage to the environment, as well as promote invention in many industries [4]. The zero-waste approach, based on minimising waste generation and fully exploiting industrial by-products, may play a significant role in addressing environmental challenges [4,5].

One of many underutilised crops with considerable potential in this regard is the genus *Pistacia* (family Anacardiaceae, order *Sapindales*) comprising 11 species found in the Mediterranean basin, the Middle East, parts of Asia, and the Americas [5,6,7,8]. The most important species, *Pistacia vera* L., is grown all over the world for its seeds, which are valued for their culinary and health properties [9]. US pistachio exports alone constituted more than 535,000 tons in 2023/24; this is 31% more than the previous year with the increase in sales attributed to increased demand in Europe and Asia [10]. As production volumes are expected to rise in the coming years, large amounts of post-harvest and post-processing waste, such as shells, hulls, leaves, resin, and pruning waste are being generated. Effective waste management is required to implement proper and ecologically acceptable waste disposal methods without affecting production sustainability [6,10,11].

Recent studies have also highlighted high content of phenolic compounds, flavonoids, carotenoids, and essential oils in different parts of the plant. These substances have strong antioxidant, antibacterial, and anti-inflammatory properties and are believed to offer protection to the nervous system [12,13,14,15,16,17,18]. As such, interest has grown in products based on pistachio waste, especially cosmetics, medicines, and food [6,12,19]. These by-products have exhibited a variety of useful applications in agriculture and environmental engineering [20,21]. For example, they can be used as an inexpensive and environmentally friendly resource for the removal of industrial pollutants, such as dyes, antibiotics, and heavy metals from wastewater [22,23,24]. Pistachio-based materials have also demonstrated potential for corrosion inhibition, biogas production, pelletisation [25,26], and the formulation of botanical insecticides and fumigants [27,28]. In agricultural systems, extracts from pistachio leaves and twigs have been found to affect plant growth and animal health, and encapsulated hull extracts can extend the shelf life of fresh produce [20,21,29,30,31].

*Pistacia* species, particularly *P. vera*, play a significant role as both economic crops and valuable bioresources. Despite these encouraging results, the use of pistachio waste is frequently confined to the local or experimental scales. Hence, there is a need to upscale biorefinery technologies, optimise extraction protocols, standardise product quality, and integrate these resources into commercial value chains [29,30,31].

This review summarises the latest research on the industrial potential of *Pistacia* spp., focusing on the utilisation of its by-products in sectors such as agriculture, food technology, cosmeceuticals, and environmental engineering. This emphasises the relevance of pistachio-derived residues in the circular economy and the opportunities they offer for sustainable innovation in crop-based industries.

## 2. Study Design

The aim of this review was to investigate the potential uses of *Pistacia* derivatives obtained from various parts of the plant across multiple industrial fields, with particular emphasis on the reintegration of agricultural residues into industrial processes.

A search was performed using the Scopus, Web of Science, and PubMed databases following the PRISMA guidelines, without applying any time restrictions, to cover the widest possible range of publications and ensure the comprehensive character of this review covering previous experience and work. Patents describing the implementation of *Pistacia* spp. were systematically searched using the Google Patents database. The search was conducted using combinations of keywords including ‘Pistacia’, ‘pistachio’, ‘Pistacia vera’, ‘bioactive compounds’, ‘extraction’, ‘pharmaceutical’, and ‘cosmetic applications’. The inclusion criterion was patents that explicitly reported the use of *Pistacia* species in technological, pharmaceutical, nutraceutical, or cosmetic applications. Patents unrelated to *Pistacia* spp. that lacked sufficient technical details or were outside the scope of application-focused innovations were excluded. Both filed and granted patents were considered and duplicate records within patent families were consolidated to avoid redundancy. The retrieved patents were further categorised based on their primary applications (e.g., medical, food, cosmetic, or industrial use) and the type of innovation (e.g., extraction methods, formulations, or bioactive compound utilisation).

**Figure 1:**
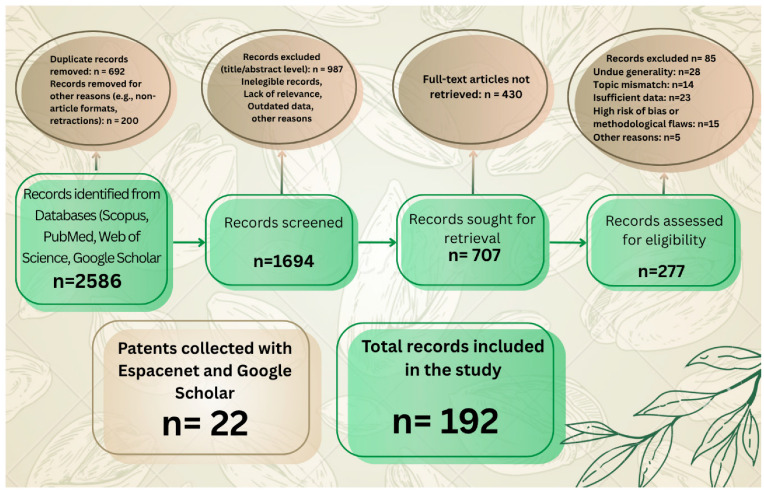
PRISMA flow diagram demonstrating the screening method and exclusion criteria. Figure was generated using © 2024 BioRender and © 2025 Canva.

References for this study were selected in July 2025 and March 2026 to ensure an updated search guarantee in the preparation process of this study. Only studies illustrating specific examples of *Pistacia* applications in broadly defined industries are included. The selection and exclusion procedures are shown in Figure 1.

The following keywords were used: *Pistacia*, *Pistacia vera*, *Pistacia atlantica*, *Pistacia chinensis*, *Pistacia falcata*, *Pistacia integerrima*, *Pistacia khinjuk*, *Pistacia lentiscus*, *Pistacia palaestina*, *Pistacia terebinthus*, *Pistacia* extracts, *Pistacia* waste, *Pistacia* in industry, *Pistacia* applications, *Pistacia* in cosmetics, *Pistacia* in material production, *Pistacia* in food preservation, adsorptive properties of *Pistacia* parts, repellent/insecticidal/antifungal/antimicrobial/anti-inflammatory/anticorrosive activity of *Pistacia*, *Pistacia* in agriculture, *Pistacia* in biofuel production, *Pistacia* in zero-waste economy, *Pistacia* in sustainable development, nanoparticles with *Pistacia*, and nanoencapsulation of *Pistacia* extracts.

The initial search was conducted by one reviewer in a leading manner (screening for duplicates, irrelevant content, and general information), after which the results were independently verified by all authors in the form of partial double-screening and agreement on proper level of correctness; the data were then compared and confirmed collectively before being organised into tables. Duplicates resulting from the multifaceted database sources utilised in the investigation were removed from the aspects of corrected/updated manuscripts and sources retreated from several databases. Duplication was performed with manual correction using DOI matching, title comparison, author list comparison, and content collation. Automation tools were not used during this process.

At each stage of writing, the information was jointly evaluated and any differences in interpretation were discussed to minimise bias and inconsistencies. The outcome measures varied between different sections and are reported in the tables. To preserve the accuracy and highlight the diversity and specificity of the included studies and measurement methods, the measurement units were not standardised or converted.

## 3. The Genus *Pistacia*: Global Relevance, Botanical Overview, and Traditional Importance

The genus *Pistacia* comprises dioecious trees and shrubs with alternate, deciduous, pinnately compound, and leathery leaves. Being xerophytic, members of this genus manifest a number of adaptive characteristics that are particularly beneficial in arid climates, such as a high density of stomata, hairs, and protective cells, which aid water retention [8,32]. Both male and female flowers are anemophilous and devoid of petals and form clusters of several hundred individual flowers [32,33]. A tree will start producing fruit approximately seven years after a 15–20-year maturation period, resulting in the formation of inedible spherical fruits, categorised as drupes. These fruits are produced collectively and consist of three layers: an exocarp, fleshy mesocarp, and bony endocarp surrounding a single nut. The exocarp changes colour from green to red or purple as it ripens [32,33].

The genus *Pistacia*, family *Anacardiaceae*, comprises 11 species with numerous varieties and subspecies, as asserted by Yi et al. (2008) [5]. Of these, *Pistacia vera* L. is the sole species to produce nuts of a sufficient size to be utilised in the food and confectionery industries [8]. The global pistachio market is characterised by the dominance of a small number of producing countries with Iran, the United States, and Turkey accounting for approximately 80% of global production in the last decade [34]. Following the recent increase in demand driven by digital marketing and social media, according to ref. [35] it is anticipated that by 2025, production in the United States (583,525 tons) will significantly surpass that of Turkey (181,536 tons) and Iran (178,756 tons), as well as China and Syria [36]. This potential significant increase in international production may result in a sudden drop in prices or increased cultivation; overproduction may present problems regarding sustainability and improper waste management [36], which can be addressed by exploring opportunities to reintroduce waste into the industry.

Nevertheless, other species and subspecies of *Pistacia*, such as *P. atlantica*, *P. chinensis*, *P. chinensis* subsp. *integerrima*, *P. khinjuk*, *P. lentiscus*, *P. mexicana*, *P. palaestina*, *P. saportae*, *P. terebinthus*, *P. texana*, *P. vera*, and *P. weinmannifolia*, have been used in oil extraction, soap production, charcoal production, varnishes, coasters, beverage production, or traditional medicine [32,37]. The *Pistacia* genus is distributed across a wide geographical area. Although *Pistacia vera* is the most widely grown species, *P. lentiscus* is also valued for its resin (mastic), which is used in traditional medicine to treat inflammatory diseases [33,37], and *P. terebinthus*, also known as the turpentine tree, is widely used in the perfume and chemical industries because of its pleasant scent [38]; all three species grow mainly in the Mediterranean basin and Asia. *P. chinensis* (Chinese pistachio) is native to China and Taiwan and is cultivated for aesthetic purposes [33,37]. *P. aethiopica* is endemic to East Africa and *P. afghanistana* to South-Central Asia. *P. atlantica* has been identified in Central Asia, [5] and North Africa, where it plays a key role in the reforestation of arid areas of Algeria [33,39]. *P. mexicana* and *P. texana* are cultivated to a limited extent in North and Central America and are threatened by habitat loss. In 2014, these species were listed on the IUCN Red List of Threatened Species as near-threatened [37,40].

*Pistacia* spp. are used in many traditional medicinal solutions. The leaves of *P. lentiscus* have been used as anti-inflammatory, antipyretic, and antiseptic agents, whereas resin has been used to treat dental, stomach, and intestinal diseases and as a brain and liver tonic. The resin of *P. atlantica* has been utilised in traditional Iranian medicine for a variety of purposes, including as a diuretic, laxative, or phlegm thinner, as well as in the treatment of gastrointestinal or kidney disorders [41]. *Pistacia vera* has been valued for its culinary properties since 7000 BC, but its resin has also been found to be useful in treating abdominal pain, diarrhoea, and haemorrhoids [41]. *Pistacia terebinthus* resin has been used as an aphrodisiac in Greece, and its fruit in the preparation of Kurdish coffee [41,42]. Furthermore, *P. vera*, *P. terebinthus*, and *P. lentiscus* have historically been used as natural fabric dyes in the textile industry [43,44].

## 4. Phytochemical Profile of Various Parts of *Pistacia* Plants

The popularity of *Pistacia* can be attributed to the composition of its nuts and other parts, which may vary between cultivation regions and species [45].

Edible kernels of *P. vera* are particularly rich in monounsaturated oleic acid and polysaturated linoleic acid, constituting approximately 80% of the total fatty acid content [45]. Pistachios are also high in protein (24.5 ± 0.4 g/100 g in Bronte Pistachio) and dietary fibre (15.3 ± 0.32 g/100 g full) and contain various trace elements (Fe, Zn, Cu, Mn) and minerals (Ca, P, K, Mg, Na). They have also been found to contain high levels of total polyphenols, mainly lutein (1.26 mg/100 g), β-carotene (0.18 mg/100 g), and γ-tocopherol (19.2 mg/100 g), as well as phytosterols, including β-sitosterol (86% of total phytosterols) or Δ5-avenasterol (6.3%). Nuts have a wide spectrum of effects and demonstrate strong antioxidant activity owing to their phenolic content [46,47].

Pistachio kernels contain negligible quantities of essential oil fractions; however, monoterpenes and sesquiterpenes, predominantly terpinolene (54.6%) and α-pinene (31.2%), were identified. Most significantly, the essential oil from *P. terebinthus* fruit demonstrated notably elevated levels of limonene (34.2%) compared to other species, *P. atlantica* oil is rich in bornyl acetate, and *P. palaestina oil* is dominated by terpenoils (E)-ocimene and sabinene [48].

In addition to its fundamental composition of cellulose, hemicellulose, and lignin, [49], pistachio nutshells contain negligible quantities of volatile compounds (e.g., 4-carene, α-pinene, or D-limonene in the case of *P. vera*), which endow them with their distinctive aroma [50]. *P. vera* shells are distinguished by high levels of anacardic acids, fatty acids (linoleic, oleic, and palmitic acids), and phytosterols (inter alia beta-sitosterol) [51]. Interestingly, the phenolic acid and flavonoid content of *P. vera* shells varies according to maturity: mature shells have higher levels of gallic acid, caffeic acid, epicatechin, syringic acid, and hesperidin, while unripe shells have higher levels of protocatechuic acid, p-hydroxybenzoic acid, p-coumaric acid, quercetin, apigenin, (+)-catechin, and kaempferol; these compounds may also be absent from mature shells. This suggests that unripe pistachio shells may have a higher biological activity [52].

Regarding volatile compounds, *Pistacia* leaves and twigs of the volatile compounds had similar general compositions, although higher levels were observed in leaves. In *P. terebinthus*, the main terpenes in leaves are bornyl acetate, phytol, α-cadinol, delta-cadinene, and α-terpineol, whereas germacrene D, beta-pinene, alpha-cubebene, and cubebol predominate in the twigs. In *P. lentiscus*, terpinene-4-ol and beta-caryophyllene were common to both leaves and twigs; higher levels of p-cymene were noted in the leaves, and sabinene, alpha-pinene, germacrene D, and limonene were found in the twigs [47,53]. The leaves were also characterised by a higher total phenolic content than the nuts, shells, stems, and roots [54,55]. *Pistacia lentiscus* fractions contained gallic acid, ascorbic acid, vanillic acid, p-coumaric acid, catechin, ferulic acid, quercetin, and naringenin. The leaf extracts demonstrated strong antioxidant activity, which was attributed to high levels of anthocyanins, tannins, and phenolic acids [55].

Pistachio resin has long been used in traditional medicine. Resin obtained from *P. lentiscus*, commonly known as “mastic” or “mastic gum”, is light-yellow and transparent when fresh, but becomes dull and brittle over time. Its aroma and fragrance are particularly prized [56]. The essential oils contain a minimum of 30 compounds, with their concentrations changing over time: fresh samples exhibit elevated levels of α-pinene and β-myrcene, while camphene is only detected in older samples as a by-product of isomerisation. Mastic oil has been found to contain α-campholene, pinocarveol, verbenone, and pinocarvone and demonstrates antibacterial activity, especially against *Aspergillus nidulans*, *Aspergillus fumigatus* and *Mucor circinelloides* [57]. Pistachio resin contains a mixture of carbohydrates, including arabinose, galactose, rhamnose, and xylose, and amino acids, such as glutamic acid, aspartic acid, serine, proline, and histidine; however, the levels of different carbohydrates and volatile compounds differ between growing regions [58].

**Figure 2:**
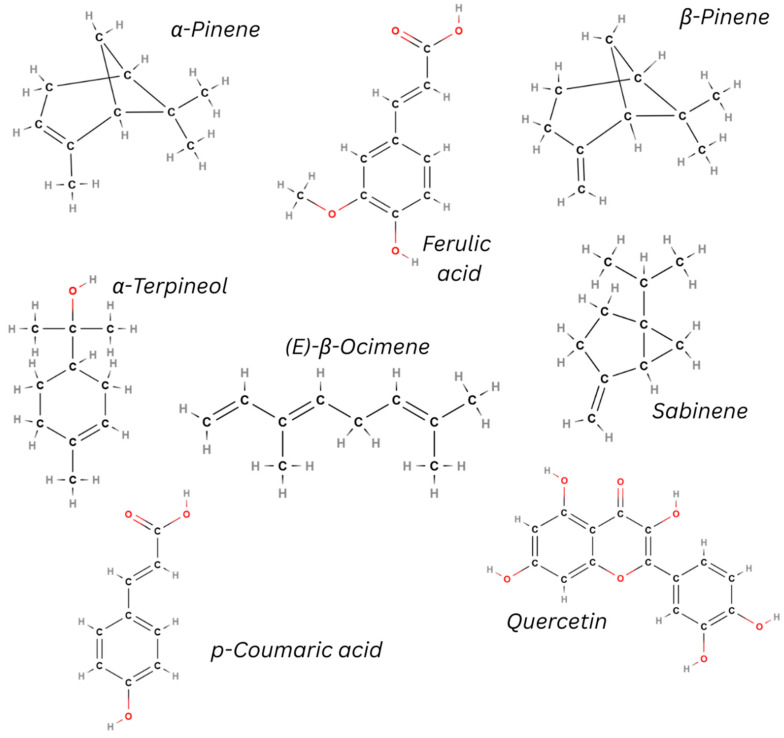
Chemical structure of selected secondary metabolites of *Pistacia* genus plants. The structures were developed using MolView software (version 2026-04-29: aaf08a83).

Selected chemical components present in various parts of the plants of the *Pistacia* genus are shown in Figure 2.

## 5. The Use of *Pistacia* Species in Agriculture

Pistachios (*Pistacia vera* L.) are cultivated worldwide as edible kernels. However, their processing generates large quantities of by-products rich in dietary fibre, polyphenols, and tannins, including the outer skin (epicarp), hulls, shells, and kernel residues. It has been proposed that they can be used as feed ingredients for ruminants, which can reduce feeding costs, improve resource efficiency, and support sustainable livestock production. Recent studies have evaluated the nutritional value of various pistachio by-products and their potential effects on growth performance, product quality, and metabolic responses in sheep, goats, and cattle. Salehi et al. [59] examined the effect of ensiled pistachio epicarp in Afshari lamb diets at 0, 8, 17, and 25% of the total ration (≈0–80–170–250 g/kg DM). Consumption affected fleece weight, wool fibre diameter, breaking load, and tenacity in a dose-dependent manner. However, the authors emphasise the importance of applying a correct dose, as condensed tannins can have beneficial effects at moderate concentrations (about 20–40 g/kg DM in the diet) but may reduce intake and digestibility at higher levels (~50–100 g/kg DM) [59].

In a short-term Latin square trial, Rezaeenia et al. (2012) evaluated pistachio hull silage as a partial replacement for corn silage in the diets of Holstein dairy cows at levels up to approximately 15% DM [60]. The consumption had no effect on milk yield, composition, or key blood metabolites, suggesting that moderate inclusion of hull silage is feasible without impairing performance [61]. Norouzian and Ghiasi assessed the replacement of alfalfa hay and beet pulp with ground pistachio by-products in the diet of Baluchi lambs at 0, 10, 20%, and 30% DM. Up to 30% inclusion had no negative effect on growth rate, carcase yield, or concentrations of Ca, Zn, Fe, and Cu in the longissimus dorsi muscle, indicating no detrimental effect on meat mineral composition [62].

The replacement of corn silage with silage made from pistachio by-products at 0, 6, 12, and 18% DM in male Holstein calves had no significant effect on the digestibility of dry matter, organic matter, or ether extract; however, protein and fibre (NDF and ADF) digestibility decreased linearly with increasing silage content. Chewing activity was also affected by some doses, with a 6% increase in the rumination time. Again, it appears that while small additions are acceptable, higher levels may limit nutrient utilisation due to their tannin content [63]. Further research in this area is summarised in Table 1.

## 6. The Use of *Pistacia* Species in Food Enhancement and Preservation

Pistachio by-products are increasingly being used in food preservation and value-added processes. For example, green pistachio shells and outer hulls are rich in phenolic compounds, flavonoids, and volatile components, with documented antioxidant, antibacterial, and antifungal properties. The development of extraction methods, nanoencapsulation, and edible coating technologies has enabled the incorporation of bioactive compounds into packaging systems and direct food applications. This extends the shelf life of the product, increases food safety, and reduces oxidation. The following studies present innovative ways of using pistachio by-products in post-harvest processing to ensure food safety and enable natural food preservation. Ebrahimian et al. evaluated the effect of adding nanoencapsulated green pistachio hull extract (PGHE) to edible coatings based on carboxymethylcellulose (CMC) and soy protein isolate (SPI) on the quality of fresh Kale Ghoochi pistachios during 18 weeks of storage at ambient temperature. At the end of the storage period, the coated samples containing 2% and 3% PGHE demonstrated significantly increased moisture content (+19.5%) and total phenolic content (+75.6%) compared with the uncoated samples, which were also characterised by a lower acid value (−41%), peroxide value (−53%), and microbial load (total viable count −44%, total faecal coliform count −35%). These findings indicate that green hull extract can extend the shelf life of pistachios by acting as a natural antioxidant and antibacterial agent [78].

Fatemi et al. investigated the antioxidant and antifungal properties of pistachio hull extract (PHE) in nanoemulsions formulated with fenugreek seed gum (FG) and whey protein isolate (WPI) for storing fresh pistachios at 4 °C for eight weeks. The extract, which is rich in phenolic compounds (~675 mg GAE/g), exhibited greater antioxidant activity than TBHQ and inhibited the growth of *Aspergillus flavus*, *A. parasiticus* and *A. nomius*. Coating containing 100 ppm PHE significantly reduced oil oxidation and mould growth, while maintaining acceptable sensory quality and moisture. The authors emphasised that pistachio hull extract could play a role in active packaging solutions [29].

Krichen et al. [79] examined the characteristics of essential oil (EO) obtained from pistachio shells during the processing. A total of 42 compounds were identified, with the main compounds being E-9-octadecen-1-ol acetate, 11-hexacosene, and 1-eicosanol. The EO exhibited notable antioxidant activity in DPPH (IC_50_ = 4.6 mg/mL), β-carotene/linoleic acid (IC_50_ = 2.7 mg/mL), and ABTS (IC_50_ = 0.2 mg/mL) assays. Strong antibacterial effects were observed against *Salmonella typhi*, *Enterococcus faecalis*, and *Escherichia coli* at a concentration of 100 mg/mL. The incorporation of the oil into minced beef significantly reduced lipid oxidation during chilled storage, suggesting its potential as a natural food preservative [79]. Similar studies are summarised in Table 2.

## 7. The Use of *Pistacia* Species in Anti-Corrosion Agents

Soltani et al. examined the use of *Pistacia khinjuk* aerial part extract in hydrochloric (HCl) and sulfuric (H_2_SO_3_) acid solutions as an ecological anti-corrosion agent for carbon steel. At a concentration of 2 g/L, the extract prevented 92.8% of corrosion in HCl and 90.3% in H_2_SO_4_ at temperatures between 25 and 65 °C. This was attributed to the physical adsorption of bioactive compounds from the extract onto the metal surface in accordance with the Langmuir adsorption isotherm. Electrochemical measurements confirmed a mixed inhibitory action, while the density functional theory (DFT) calculations confirmed an interaction between the key components of the extract and the steel surface [107].

In turn, Dahmani et al. demonstrated that the essential oil of *Pistacia lentiscus* (Pil) is an excellent inhibitor of copper corrosion in 0.5 M H_2_SO_4_. They showed that inhibition efficiency (IE%) increases with inhibitor concentration, reaching 94% at 2.0 g/L. The copper surface immersed in the solution was characterised by scanning electron microscopy (SEM)/energy-dispersive spectroscopy (EDS), atomic force microscopy (AFM), and contact angle (θ) measurements. Experimentally obtained corrosion inhibition efficiency correlated well with theoretical calculations (DFT and MC simulations) [108].

Similarly, Kaur et al. reported that *Pistacia integerrima* gall extract demonstrated promising corrosion inhibition in mild steel in 1 M H_2_SO_3_. Increasing the inhibitor concentration reduced the corrosion rate and improved the efficiency, with a maximum inhibition efficiency (92.19%) obtained at an inhibitor concentration of 2000 mg/L. SEM and AFM analyses confirmed the presence of a protective film, and DFT calculations indicated that pistacophenyl ether extract demonstrated the highest adsorption energy and predicted inhibition strength, followed by pistifloroglucine ether and naringenin. The authors concluded that *P. integerrima* gall extract is a promising green corrosion inhibitor for acid pickling and cleaning applications [109]. Other studies are summarised in Table 3.

## 8. The Use of *Pistacia* Species in the Cosmetics Industry

Pistachio kernels, shells, and green husks are becoming increasingly important in cosmetics and hygiene industries. These by-products are a valuable source of fatty acids, phenols, and aromatic compounds with antibacterial, anti-inflammatory, and antioxidant properties and have been increasingly exploited by modern extraction techniques and cosmetic formulations such as rinses and emulsions.

A study based on liquid chromatography coupled with high-resolution mass spectrometry (HPLC-HRMS) by Benalia et al. identified a number of alkyl salicylates in lentisk oil (*Pistacia lentiscus* L.), with the following predominant compounds: 3-pentadecylsalicylic acid, 5-pentadecylresorcinol, and 6-heptadecylsalicylic acid. These substances demonstrated low cytotoxicity against human skin fibroblasts (HNDF) at concentrations of up to 40 µM. These results suggest that *L. pistachio* oil can be used as an anti-inflammatory and non-irritating ingredient in dermocosmetic formulations [116].

Arami et al. conducted a triple-blind clinical trial to evaluate the effectiveness of a mouthwash containing *Pistacia atlantica* var. mutica resin to reduce plaque and subgingival microorganisms. After four days, the number of bacteria in the test group decreased from 2.17 × 10^6^ to 7.25 × 10 CFU/mL. This was comparable to the efficacy of chlorhexidine (9.91 × 10^3^ CFU/mL) and significantly better than that of the placebo. The preparation exhibited antibacterial activity and had low potential to cause irritation, making it a promising ingredient for natural oral hygiene products [117].

A study based on inductively coupled plasma optical emission spectrometry (ICP-OES) examined the trace elements present in raw *Pistacia terebinthus* L. fruit as well as in soap and gum extracts. Thirteen elements were identified, including zinc, iron, copper, manganese, and magnesium, with the highest concentrations found in the gum. Hence, pistachio fruit preparations may be a natural source of micronutrients with potential applications in skincare and dermatological products [118]. Further studies are presented in Table 4.

## 9. Use of *Pistacia* Species for the Production of Composite Materials

Pistachio by-products are increasingly being recognised as valuable raw materials for the production of composite materials and pharmaceuticals. Their bioactive compounds can be used to enhance the mechanical and thermal properties and to improve controlled drug release. Salazar-Cruz et al. developed polypropylene (PP) composites by adding chemically modified particles of the *Pistacia vera* nutshell particles. The shells were modified to remove lignin and hemicellulose (as confirmed by TGA and FTIR), which increased thermal stability and induced nucleation. Higher crystallinity, melting temperature (Tm), and elastic modulus were observed, particularly at a dose of 4 phr [138].

Pączkowski and Gawdzik described the preparation of eco-friendly composite from thermosetting resins (polyester and vinyl ester) incorporating pistachio shells as filler. The procured specimens were subjected to accelerated ageing and exposure to solvents, and the results showed UV radiation absorption and potential for use as a charring agent. The addition of pistachio shell waste also caused an increase in water sorption and a subsequent plasticisation effect [139].

Morkhade examined the use of natural mastic gum (*Pistacia lentiscus*) for microencapsulation to create pharmaceutical matrices for model drugs such as diclofenac and diltiazem. Microspheres with diameters of 22–62 µm and loading efficiencies of 50–87% were produced. With the correct gum-to-drug ratio, these microspheres achieved prolonged release of up to 12 h. Tablets containing 30% mastic gum had extended release times of 8–11 h [140].

Najafiasl et al. used oil extracted from the leaves and unripe fruits of *Pistacia atlantica* as a natural antioxidant and structural agent in electrospun polyvinyl alcohol (PVA)/sodium alginate (SA) nanofibres. The addition of excess oil (PAO) improved the mechanical properties; nanofibres containing 1.5% (*w*/*v*) PAO exhibited 3.5 times increase in tensile strength compared with those without oil. PAO also increased the thermal stability of the nanofibres (the DTG showed a shift in degradation to a higher temperature) and their antioxidant activity (the IC_50_ in the DPPH test was 7.26 mg/mL). Additionally, a mass transfer model based on two-film theory was developed to accurately predict the PAO release kinetics from the fibres under different pH conditions [141]. Other studies are presented in Table 5.

## 10. Applications of *Pistacia* Species in the Adsorption Removal of Environmental and Chemical Pollutants

Pistachio by-products, particularly shells, show great promise as environmentally friendly substrates for producing adsorbent materials. Owing to their porous structure and surface-active compounds, they can effectively remove contaminants, such as organic dyes and heavy metals, from water. The work presented below demonstrates the innovative use of pistachio shells in adsorption and photocatalysis for environmental protection. Şentürk and Alzein used acid-activated pistachio shells (with 10 N H_2_SO_3_) as adsorbents for acid violet 17 dye. Up to 93.04% removal was achieved, with adsorption following pseudo-second-order kinetics. Adsorption was characterised by a Temkin isotherm and a Langmuir isotherm q(max) value of 26.45 mg/g. The process was endothermic and reversible, with 97.33% desorption achieved in 0.2 M NaOH. This economical bioadsorbent has practical applications in the treatment of industrial wastewater [175].

Pistachio shell powder was used to remove the (CV) dye, and the conditions were optimised using the Taguchi method. At a pH of 6, CV concentration of 10 mg/L, contact time of 360 min, and dosage of 4 g/L, and efficiency of 93.6% ± 0.1% was achieved. The adsorbent concentration had the greatest impact on the result (33.17%). This approach is an inexpensive method for removing dyes from water [176].

Bazrafshan et al. used ZnCl_2_-activated pistachio shell ash to adsorb the methylene blue dye. The maximum adsorption capacity was found to be 155.6 mg/g at a pH of 8 and at a temperature of 20 °C, with a dye removal rate of 97.25%. The data best fitted the Freundlich model (R^2^ = 0.9522). Hence, pistachio shell ash can effectively remove cationic methylene blue dye from coloured wastewater [177].

Naeimi et al. synthesised an innovative bentonite/cellulose@PbO bio-nanocomposite; briefly, the study used an environmentally friendly approach that employed *Pistacia atlantica* leaf extract as a reducing and stabilising agent. This extract played a pivotal role in the formation of lead oxide nanoparticles within the bentonite–cellulose matrix. The resulting nanocomposite exhibited exceptional photocatalytic efficiency, achieving 98.3% degradation of ciprofloxacin within 60 min under visible light irradiation. The obtained material exhibited high reusability over five cycles with minimal loss of activity. Hence, *Pistacia atlantica* leaf extract is a promising candidate for environmental remediation, particularly in the treatment of wastewater contaminated with pharmaceuticals [178].

In another study, powder from pistachio shells, along with walnut, hazelnut, almond, apricot stone, and shell materials, was carbonised at 800 °C and evaluated under optimised conditions to remove copper (Cu^2+^), lead (Pb^2+^), cadmium (Cd^2+^), and zinc (Zn^2+^). Carbon obtained from pistachio shells exhibited a moderate adsorption efficiency, achieving over 80% removal of Cu^2+^ from synthetic wastewater and over 95% removal from real industrial wastewater samples from copper production. Among all tested materials, pistachio shell carbon showed the lowest surface area (635 m^2^/g); nevertheless, it still demonstrated a good removal potential. Although pistachio shells demonstrated less capacity than other nutshells (e.g., almond and hazelnut), pistachio shell-based carbon can still serve as an inexpensive and sustainable bioadsorbent for treating metal-contaminated industrial effluents, especially copper and zinc [179].

Similar results were obtained by Adhikari et al., who demonstrated that activated carbon derived from pistachio shells effectively removes pollutants from the Bagmati River. The material was prepared through chemical activation with H_3_PO_4_ followed by carbonisation at 400 °C. Over 80% of heavy metals (e.g., Fe and Cr), phosphates, and nitrates reduced water hardness, turbidity, and conductivity. Following the treatment, the water quality met the WHO standards, thereby confirming the suitability of pistachio shells as an inexpensive and effective material for treating surface water [180].

El-Azazy et al. assessed the effectiveness of various agro-waste materials, including pistachio shells, for removing sarafloxacin from water. Pistachio shells demonstrated excellent adsorption capacity, with a value of qmax = 11.68 mg/g (Langmuir model), ranking second only to activated carbon and carbon nanotubes. Adsorption proceeded according to pseudo-second-order kinetics and was controlled by chemisorption. These results confirm that pistachio shells can be used as environmentally friendly and inexpensive sorbents to remove antibiotics from contaminated water [181]. Further studies are presented in Table 6 and Table 7.

## 11. Utilisation of *Pistacia* Species in the Development of Biofuels

Oil plants and their waste are becoming increasingly important in the production of second-generation biofuels, driven by the increasing demand for renewable energy sources and the need to reduce greenhouse gas emissions. Pistachio fruit oil can be converted to biodiesel via transesterification or catalytic pyrolysis, while shells and other lignocellulosic wastes can be used in inter alia pyrolysis, carbonisation, and biochar production. This approach reduces biomass waste and increases the added value of the pistachio production chain by integrating it into the biofuel sector. Unlike edible oils, raw materials derived from wild or semi-wild plants do not compete in the food chain, making them a more sustainable option. Subsequent studies have demonstrated the potential of pistachio fruit oil as an inexpensive and biodegradable energy source [225].

Ahanchi et al. examined the technical and environmental aspects of pistachio shell extract as a natural substitute for propyl gallate in rapeseed biodiesel. It was found that 2500 ppm of the bio-antioxidant and 250 ppm of the synthetic antioxidant were required to extend the induction period of the tested biodiesel from 1.53 h to three hours, in accordance with the biodiesel oxidative stability specification requirements. Therefore, using agricultural waste to produce natural antioxidants, such as pistachio shell extract, appears to be a promising strategy for enhancing the environmental and health benefits of biodiesel [225].

Kar et al. analysed the impact of various pyrolysis parameters, including the final temperature, heating rate, particle size, and nitrogen flow rate, on the yield of *Pistacia terebinthus* L. (terebinth) fruit as a bio-oil source. The maximum bio-oil yield of 58.99% by weight was obtained, which was almost equal to that of diesel fuel. In addition, when produced under optimal conditions, bio-oil can serve as a raw material for producing diesel-like fuels or refined chemicals [226].

Another study investigated the effect of the pyrolysis temperature on the yield and composition of products obtained from the slow pyrolysis of pistachio shells in a fixed-bed reactor under atmospheric pressure. Temperatures ranging from 300 to 700 °C were applied. The maximum liquid yield of 20.5% was achieved at temperatures of approximately 500–550 °C. The results indicated that the liquid and solid products obtained from pistachio shells were comparable to those obtained using high-quality conventional fuels [227].

In addition, da Silva et al. demonstrated the usefulness of pistachio shells in combustion processes and the possibility of simulating decomposition curves using data obtained using the isoconversion method, presenting a satisfactory approximation using simple numerical methods [228]. Further research is presented in Table 8.

## 12. Insecticidal, Repellent, and Fumigant Applications for *Pistacia* spp.

Although *Pistacia* spp. are mainly known to be a source of highly nutritious seeds, they also show bioactive potential for protection against insects. The components of pistachio leaves, fruits, resins, and shells can act as pest repellents. The use of plant extracts as natural repellents is in line with current trends in plant bioprotection and the production of environmentally friendly protective preparations.

Lebbal et al. investigated the effects of extracts and oils from *Pistacia lentiscus* on pink aphids (*Dysaphis plantaginea*). The results showed that among the tested aqueous extracts, the 9% concentration was the most toxic to aphids, with a mortality rate of 50.77%, while the 5% concentration was the most effective at repelling them, with a repulsion rate of 53.33%. Regarding essential oils, a concentration of 10,000 ppm proved to be the most effective, resulting in a mortality rate of 71.13% and repulsion rate of 66.67% [237].

Another study evaluated the repellent effect of *Pistacia lentiscus* essential oil (PEO). The results showed that the oil exhibited a broad spectrum of repellent activity against target pests, with RD_50_ values ranging from 0.010 to 0.037 μL cm^−2^. Therefore, it is a potentially effective and safe pest control agent [238].

Ravan et al. demonstrated the insecticidal activity of *Pistacia khinjuk* leaf extract against *Tribolium confusum* and *Oryzaephilus surinamensis*, with respective LC_50_ values of 9.32 and 5.47 mg ml^−1^. The plant extract contained a number of compounds with insecticidal properties, including spathulenol (2.07%), myrcene (2.03%), p-cymene (1.67%), apiol (1.61%), borneol (0.79%), and pulegone (0.44%), confirming that *P. khinjuk* extract has the potential to control insect pests [239].

Another study demonstrated the insecticidal activity of essential oils extracted from the aerial parts of *Pistacia lentiscus* (PLEO) against *Tribolium castaneum*. The main components of these oils were α-myrcene, limonene, and α-pinene. Contact toxicity testing revealed that PLEO exhibited potent insecticidal properties against mealworm beetles, suggesting its potential as a repellent [240]. Further details are presented in Table 9.

## 13. The Use of Different *Pistachio* spp. in Combination with Nanotechnology in Industry

One particularly interesting area of research is the use of nanomaterials in combination with plant waste fractions, which are typically reserved for use in animal feed or fuel generation. By-products generated during processing, such as pomace and shells, contain high levels of phenolic compounds, proteins, and polysaccharides, which make them valuable raw materials for the production of nanoparticles, biopolymers, and functional coatings. Nanotechnology is used in the pharmaceutical, food, and cosmetic industries to improve the bioavailability of active ingredients, create materials with controlled mechanical and barrier properties, and develop nanocomposites with better durability and biological activity. These activities improve the sustainable use of resources and increase the value of the processing chain [254]. Some uses of pistachio pomace in nanotechnology are summarised in Table 10.

**Table 10:** Nanotechnology-related findings on potential application of *Pistacia* sp.; synthesis, utilisation and formulation.

*Pistacia* Species	Type of Material	Part of Plant	Preparation Method	Effects	Reference
*Pistacia vera* L.	Silver nanoparticles	Hull	Extracts of *P. vera* and *S. ebulus* were used to obtain silver nanoparticles by reducing AgNO_3_ in their presence.	The produced nanoparticles exhibited high antibacterial properties against both Gram-positive and Gram-negative bacteria, with the highest activity measured against *S. aureus* and *E. faecalis.* They were also found to possess strong anticancer effects on MCF-7 and AGS cancer cell lines.	[254]
*Pistacia khinjuk* Stocks	Silver nanoparticles	Leaf	*P. khinjuk* leaf extract was added to a silver salt solution in order to create the nanoparticles.	*P. khinjuk* leaf extract has proven to be an effective, natural, and nontoxic reducing and stabilising agent with potential to replace harmful chemicals typically used in the nanoparticle synthesis process. The created compound showed antimicrobial and antioxidant activities, alongside strong cytotoxicity against some leukaemia cancer cells.	[255]
*Pistacia vera* L.	Gold nanoparticles	Hull	*P. vera* extract was used as a reducing agent in the synthesis of gold nanoparticles by introducing it to a HAuCl_4_·3H_2_O solution.	Gold nanoparticles synthesised using the *P. vera* extract showed high antibacterial properties against drug-resistant and reference pathogens, good antifungal activity against some *Candida* genus fungi, as well as notable anticancer effects.	[256]
*Pistacia atlantica* Desf.	Calcium oxide nanoparticles	Leaf	To synthesise calcium oxide nanoparticles *P. atlantica* extract was mixed with a calcium chloride solution and treated with NaOH.	The process successfully yielded spheroid calcium oxide nanoparticles with a typical size in the range of 30–100 nm, proving that the plant extract can be used in a simple and eco-friendly synthesis.	[257]
*Pistacia vera* L.	Cerium oxide nanoparticles	Pericarp	*P. vera* pericarp essential oil (PVEO) was used in the synthesis of cerium oxide nanoparticles as a stabilising agent.	Cerium oxide nanoparticles of desired physical and chemical properties were obtained with the usage of PVEO. They exhibited strong cytotoxic effects against breast and prostate cancer cells.	[258]
*Pistacia* sp.	Silver nanoparticles	Bark	AgNO_3_, chitosan-polyvinyl alcohol, and *Pistacia* tree bark extract were used for the synthesis of silver nanoparticles.	The usage of *Pistacia* tree bark extract has proven successful in the synthesis of nanoparticles, with the resulting product showing significant anticancer properties.	[259]
*Pistacia vera* L.	Organic nanocomposite	Hull	The extract of *P. vera* hull was used as a stabilising and reducing agent in the synthesis of copper nanoparticles via a reaction with copper (II) acetate monohydrate.	*P. vera* hull has proven to be effective in synthesising the nanocomposite with the process itself remaining simple. The resulting material has exhibited high antimicrobial and antifungal effects, even in low concentrations.	[260]
*Pistacia khinjuk* Stocks	Iron oxide nanoparticles	Leaf	A FeCl_3_⋅6H_2_O solution was mixed with the *P. khinjuk* solution to carry out the synthesis of the nanoparticles.	The created compound demonstrated a wide range of properties with potential uses in biomedical and environmental fields, including antibacterial and antioxidant effects, high stability, and photocatalytic degradation of the Reactive Black-5 dye.	[261]
*Pistacia khinjuk* Stocks	Nanoemulsion	Fruit	A nanoemulsion was prepared using the *P. khinjuk* balango and fenugreek seed gum extracts.	The resulting nanoemulsion was found to have high phenolic content and strong antioxidant activity. As a result, when the mixture was added to sunflower oil, it has shown improved stability with lower rates of oxidation.	[90]
*Pistacia atlantica* Desf. subsp. *kurdica* (Zohary) Rech. f.	Oleo-gum-resin-loaded electrospun nanofibers	Resin	The electrospinning technique was used to create polyvinyl (PVA) and *P. atlantica* subsp. *kurdica* (PKA) nanofibres. The process was being carried out with different proportions of substrates and with differences in conditions in order to acquire optimal properties of the nanofibres.	The optimal conditions for the process were found at 30:70 *w*/*v* ratio PKA gum, with the addition of the gum decreasing surface tension of the polymer solution and causing longer and thinner fibres to form. The material was found to contribute to wound healing by leading to considerable wound size and tissue damage decrease, compared to both untreated and conventionally treated control groups.	[262]
*Pistacia chinensis* Bunge	Gold nanoparticles	Seed	*P. chinensis* extract was used alongside gold salt (HAuCl_4_) in different proportions to synthesise the nanoparticles by mixing the solutions of the substances.	The *P. chinensis* extract was proven suitable for the synthesis of gold nanoparticles, with the final product demonstrating high urease and carbonic anhydrase inhibition capabilities, while remaining stable and spherical in shape.	[263]
*Pistacia integerrima* J.L.Stewart ex Brandis	Gold nanoparticles	Gall	A solution containing gold ions was exposed to *P. integerrima* gall extract, causing the formation of nanoparticles via the reduction in the metal.	Using the plant extract has successfully enabled the synthesis of nanoparticles, with the resulting compound exhibiting high stability when exposed to different pH levels, NaCl concentrations, as well as elevated temperatures. Nanoparticles have also shown antifungal, muscle relaxant, and anti-nociceptive properties.	[264]
*Pistacia* sp.	Edible coatings	Hull	Pistachio green hull extract (PVGE) was encapsulated in edible coating comprising carboxymethyl cellulose and soy protein isolate at different *w*/*v* proportions of PVGE.	Coatings prepared with the plant extract demonstrated improved moisture retention, as well as antifungal and antimicrobial effects, while also inhibiting oxidation reactions in stored raw pistachios.	[78]
*Pistacia atlantica* Desf.	Magnesium oxide and silver nanoparticles	Leaf	Silver and magnesium oxide nanoparticles were prepared through a reaction of *P. atlantica* leaf extract with silver and magnesium nitrate, respectively.	Synthesis reactions were successful in both cases and the created compounds were found to possess significant antibacterial effects, as well as strong photocatalytic properties.	[265]
*Pistacia atlantica* Desf.	Chitosan nanoparticles in polyvinyl alcohol fibres	Fruit	*P. atlantica* oil was encapsulated into chitosan nanoparticles with sodium tripolyphosphate as a crosslinker. The resulting substance was embedded into polyvinyl alcohol nanofibres.	The encapsulated *P. atlantica* oil embedded in nanofibres demonstrated controlled release of bioactives, high biocompatibility demonstrating potential in wound healing and skin care applications.	[126]
*Pistacia vera* L.	Nanoliposomes	Hull	Pistachio green hull extract was encapsulated in nanoliposomes; different concentrations of phenolic compounds were also added.	The formulation demonstrated high antioxidant, antifungal, and antibacterial properties in mayonnaise, making it a viable option as a bio-preservative.	[86]
*Pistacia atlantica* Desf.	Nickel oxide nanoparticles	Leaf	Nickel oxide nanoparticles were synthesised by mixing *P. atlantica* leaf extract with a nickel(II) nitrate solution.	The process of synthesis was successful, showing how the plant extract can replace potentially dangerous or expensive chemicals as a reducing agent, with the resulting nickel nanoparticles being a potent and reusable nanocatalyst for other processes.	[266]
*Pistacia lentiscus* L.	Nanoemulsions	Oil	The nanoemulsion was created as a combination of *P. lentiscus* oil, water phase, and a surfactant.	Antibiotic nanoemulsion demonstrated increased antimicrobial activity, suggesting potential incorporation in enhanced antibiotic medicines.	[267]
*Pistacia atlantica* Desf.	Palladium nanoparticles	Fruit	*P. atlantica* fruit extract was used in an environmentally friendly and fast synthesis process of palladium nanoparticles.	Plant extract proved suitable for the synthesis process without the need for a template, capping agents, or any extra surfactants, yielding spherical palladium nanoparticles of the desired structure and stability.	[268]
*Pistacia palaestina* Boiss.	Silver nanoparticles	Fruit	Silver nanoparticles were created with *P. palaestina* fruit extract as the reducing agent.	Nanoparticles spherical in shape were successfully synthesised and an ointment prepared with them was shown to accelerate wound healing in tested rats.	[269]
*Pistacia atlantica* Desf.	Silver nanoparticles decorated on multi-walled carbon	Leaf	The surface of multi-walled carbon nanotubes was modified using an extract of *P. atlantica* leaves and used for in situ reduction and immobilisation of silver nanoparticles.	The usage of the plant extract was successful, and the resulting substance can be utilised as a heterogenous catalyst for degrading organic dyes, while remaining cost-effective.	[270]

**Figure 3:**
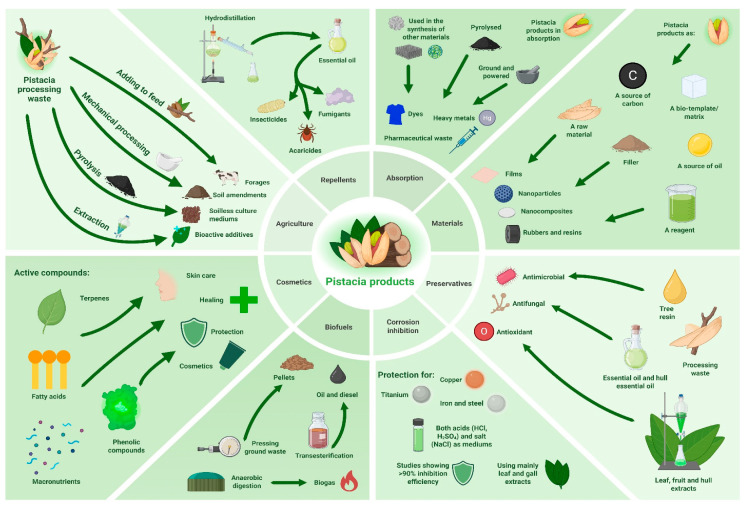
Pictorial summary of utilisation of *Pistacia* plants in different sectors of the industry. Figure was generated using © 2024 BioRender and © 2025 Canva.

The applications of *Pistacia* sp. in various industrial production fields are summarised in Figure 3.

## 14. Patents Regarding the Use of *Pistachio* spp.

The growing demand for high-value products derived from *Pistacia* spp. has led to increased research and development activities, as well as patent applications. A patent provides an inventor with the right to use, produce, or sell an invention for 20 years. By virtue of patents, entrepreneurs and inventors are at liberty to guard such technologies from emulation, thereby augmenting their competitive advantage and facilitating the recouping of expenses incurred in research and implementation [271,272]. They thus encourage greater investment in more efficient, environmentally friendly, and profitable production processes, such as automated pistachio shelling lines that minimise kernel damage and devices for sorting by size and quality (e.g., eliminating unripe or empty fruits). Pistachio waste can be used in technologies that minimise waste. It can also be used as biomass, biofuel, reinforcing material, or raw material for new products. Furthermore, they can be used in packaging systems to ensure longer freshness and visual appeal [273,274,275].

Some example patents are shown in Table 11.

## 15. Conclusions, Limitations, and Future Perspectives

Pistachio by-products are valuable and underused sources of bioactive compounds, structural biopolymers, and renewable fillers, with applications in agriculture, food technology, cosmetics, pharmaceuticals, and environmental engineering. Their antioxidant, antimicrobial, antifungal, and corrosion-inhibitory properties and their potential in nanotechnology and composite development underline their multifunctionality and capacity to support sustainable industrial innovation. The reintegration of pistachio residue into value chains is in line with the principles of a circular economy and can effectively reduce the environmental problems associated with large-scale nut production.

However, the broader industrial application of pistachio by-products is limited by several factors. First, the composition of raw materials differs depending on the cultivar, region, and processing conditions, and there are no standardised protocols for extraction and characterisation, making it difficult to scale up production and obtain reproducible results. In addition, the economic viability of these systems is uncertain, as most studies are restricted to laboratory or pilot scales, with limited incorporation into commercial supply chains. Furthermore, a thorough investigation is required regarding the sensory acceptance of nanomaterials in food applications, their cytotoxicity in biomedical applications, and their long-term stability before full industrial deployment can be considered.

The development of new uses for pistachio by-products will be driven by improvements in biorefinery technologies and expansion of green extraction processes. These processes must maintain bioactivity while ensuring that they can be reproduced reliably and economically viable. A particularly promising approach involves the use of *Pistacia*-derived fractions for the development of multifunctional nanocomposites, bio-packaging, and pharmaceutical carriers. Integration with nanotechnology, including the encapsulation of extracts and design of biochar-derived nanostructures and biosynthesized nanoparticles, has potential applications in food preservation, environmental remediation, and advanced materials. Furthermore, combining pistachio residue with other agro-industrial waste products could create new opportunities for the development of hybrid bio-based products.

## Figures and Tables

**Table 1:** Potential for utilisation of *Pistacia* species in agriculture.

*Pistacia* Species	Part of Plant	Application Type	Target Product/Activity	Obtaining Method, Application, and Testing	Found Usage Benefits	Reference
*Pistacia* sp.	Hull, twigs, leaves, shells	Forage	Saanen dairy goats	Three types of diet were tested on goats; a control diet with standard alfalfa hay, a diet with 30% pistachio by-products (PBPs), and a diet with 30% PBPs with added polyethylene glycol (PEG-4000).	The usage of PBPs containing forages did not alter milk yield or milk composition and increased the concentration of trans-C18 fatty acid. The concentrations of the major fatty acid classes remained within the same ranges.	[64]
*Pistacia vera* L.	Seed coat	Nanofertiliser	Eggplant	Silver nanoparticles were synthesised using the extract of *P. vera* seed coat waste; their effects were tested by spraying them on eggplants as a suspension.	The eggplants sprayed with the nanoparticle suspension exhibited increased growth, as well as higher chlorophyll and carotenoid contents.	[65]
*Pistacia atlantica* Desf. subsp. *kurdica* (Zohary) Rech. f.	Fruit and root extracts	Herbicide	-	Ethanolic fruit and root extract of *P. atlantica* trees was obtained via the Soxhlet method, with the solvent being expelled with a vacuum rotary evaporator. The substances were then tested for herbicidal properties.	Compared to the root, the fruit extract exhibited higher radical scavenging and antibacterial effects, with both extracts demonstrating significant herbicidal effects on the growth and germination of seedlings.	[66]
*Pistacia* sp.	Hull	Feed	Ruminant animals	Different varieties of pistachio hulls were tested for dry matter, ash, nitrogen, and condensed tannins contents, as well as in vitro gas production.	The pistachio hulls generally have the potential for meeting the minimum protein requirements for ruminant animals and are found to be digestible enough to be considered of acceptable quality as ruminant feed.	[67]
*Pistacia* sp.	Shell	Soil amendment	Bell pepper	Pistachio biomass pyrolysis was carried out at different temperatures, establishing 450 °C as optimal for soil amendment. The pyrolysis product (biochar) was added to soil to test its effects.	The usage of biochar lowered soil pH and increased nitrogen and organic carbon contents compared to unamended soil. With higher biochar content, the plant exhibited better fruit yield and higher fruit quantity.	[68]
*Pistacia* sp.	Dehulling waste	Soil amendment—compost	-	Pistachio dehulling waste (PW) was crushed and mixed with dehydrated sewage sludge; date palm straw (PS) mixed with sludge was also tested.	The PW compost showcased higher maximum temperature and larger loss of moisture but also higher toxicity compared to the PS alternative, as well as carbon-to-nitrogen ratio within the standard range.	[69]
*Pistacia* sp.	Shell	Soil amendment	Eggplant	Biochar was produced from pistachio shells via pyrolysis and mixed with cow manure at different proportions. The resulting mixtures were tested for soil amendment effects.	The addition of the biochar mixed with manure has increased plant height and leaf size, chlorophyl content, and plant biomass, while also reducing water and nutrient loss.	[70]
*Pistacia* sp.	Wood vinegar	Soilless culture mediums	Cucumber	The effects of different culture media and pistachio wood vinegar on cucumber seedling growth were tested under greenhouse conditions.	Among many improvements, shoot fresh mass, stem diameter, leaf area, root dry mass, and relative water content were all significantly increased in the seedlings treated with 0.5% wood vinegar and grown in date palm compost-vermicompost.	[71]
*Pistacia vera* L.	Processing waste (leaves, twigs, seed coats, kernels)	Bioactive additives including pesticides	-	The *P. vera* waste was dried, decocted, and tested for nematicidal activity, cholinesterase inhibition, antioxidant capacity, and phytotoxicity, in addition to chemical profiling.	The waste was identified to contain several bioactive compounds, demonstrated high antioxidant effects, and showed nematicidal activity and cholinesterase inhibition, while causing low phytotoxic effects.	[72]
*Pistacia* sp.	Processing waste (small branches, hulls, and clusters)	Soil amendment—vermicompost	Saffron	Field experiments based on a factorial randomised complete block design were carried out. Pistachio waste was used as vermicompost and the tests were carried out on two farms during multiple growing seasons.	Increased available phosphorus, decreased soil pH, better microbial respiration, and microbial biomass, alongside augmented proline content, flower number, and corm growth were all observed as benefits of using the pistachio by-products vermicompost.	[73]
*Pistacia* sp.	Processing by-products (leaf, twig, hull; deduced from a picture, branch also mentioned)	Soil amendment—compost	Saffron	In a greenhouse environment, the effects of composted pistachio residues on saffron growth were tested via an experiment based on completely randomised design.	The compost was shown to increase the number of daughter corms, decrease pH, and improve nutrient availability when applied.	[74]
*Pistacia* sp.	Processing waste	Soil amendment—compost	Saffron	An experiment based on randomised complete block design was carried out to test the effects of pistachio by-product compost and foliar spraying on saffron growth.	The use of pistachio by-product compost has increased the minimum number of saffron flowers by 27% and the stigma weight by 31%, compared to samples without fertiliser. When used alongside foliar spraying, the plants’ nutrient and bioactive compound content improved.	[75]
*Pistacia* sp.	Processing waste (cluster and green pistachio hull)	Soil amendment	*Pistacia vera* L.	Based on factorial and completely randomised design, an experiment was carried out to test the effects of pistachio by-products on plant growth.	The application of pistachio by-products increased leaf, stem, and root nutrient content of plants, while reducing negative effects of salinity on plant growth.	[76]
*Pistacia* sp.	Processing waste (shell, cluster)	Soil amendment—compost/vermicompost	-	Pistachio waste and cow manure were used to create compost via pre-composting and vermicomposting stages. The final product was assessed for its quality and properties.	The obtained compost was granular, odourless, nutrient-rich, and homogenous and demonstrated a superior chemical composition to the samples before processing.	[77]

**Table 2:** Potential for utilisation of *Pistacia* species in food enhancement and development of food preservatives.

*Pistacia* Species	Part of Plant/Material	Preservative Function	Preservative Target	Obtaining Method	Results	Reference
*Pistacia atlantica* Desf. subsp. *kurdica* (Zohary) Rech. f.	Hull essential oil	Antifungal	Strawberry	*P. atlantica* subsp. *kurdica* hull essential oil was encapsulated in chitosan nanoparticles using the emulsion–ionic gelation method.	The formed nanoparticles had an antifungal effect against Botrytis cinerea, postponing the spoilage process by 8 days for strawberries stored at 4 °C and greatly reducing the occurrence and severity of grey mould disease.	[80]
*Pistacia terebinthus* L.	Resin	Antimicrobial, probiotic preservation	Yoghurt	*Lactobacillus casei* cells were immobilised using the resin and added to adjuncts during yoghurt production.	With the addition of resin, the viability of embedded *L. casei* was sustained for 60 days of storage at 4 °C and the spoilage activity of microorganisms was reduced, with no pathogens such as *Staphylococci*, *Enterobacteriaceae*, or *Salmonella* detected.	[81]
*Pistacia vera* L.	Hull extract	Antimicrobial	Minced beef	An aqueous *P. vera* hull extract was prepared at different concentrations and added to minced beef before storage.	The meat with added extracts, especially the highest tested concentration of 0.625%, demonstrated much lower microbial counts. Additionally, the used extracts also showed significant antioxidant effects and improved sensory characteristics, such as flavour, odour, and appearance of the product.	[82]
*Pistacia lentiscus* L.	Essential oil	Antimicrobial	Ice cream, fruit juices	Essential oil was extracted from *P. lentiscus* and used as antimicrobial agent. *Fortunella margarita* was also tested.	*P. lentiscus* essential oil effectively inhibited the growth of all tested microorganisms (including *E.coli* and *S. cerevisiae*), reduced the bacterial counts in ice cream, and extended the shelf life of fruit juices when combined with *F. margarita* essential oil.	[83]
*Pistacia lentiscus* L.	Fruit and leaf extracts	Antimicrobial and antioxidant	Capriot sausages	*P. lentiscus* leaf and fruit extracts were prepared using different solvents which were later evaporated.	The extracts showed good antimicrobial effects when added to sausages, especially during the first days of storage, as well as significant antioxidant properties.	[84]
*Pistacia vera* L.	Green hull extract	Anti-browning agent	Button mushrooms	*P. vera* green hull aqueous extract, sodium metabisulfite and ascorbic–citric acids were used on post-harvest button mushrooms.	Mushrooms treated with *P. vera* green hull extract were found to exhibit improved qualities, including the highest firmness, and increased phenolic content. The extract also showed significant tyrosinase inhibition, indicating good anti-browning effects.	[85]
*Pistacia vera* L.	Green hull extract	Antimicrobial and antioxidant	Mayonnaise	Pistachio green hull extract was encapsulated into nanoliposomes and added to freshly prepared mayonnaise.	The usage of the prepared bio-preservative was shown to reduce oxidation and microbial spoilage in the samples, improve the retention and gradual release of phenolic compounds during storage, reduce the negative effects of free phenolic compounds on sensory qualities, and overall perform similarly to synthetic preservatives.	[86]
*Pistacia atlantica* Desf.	Kernel oil	Antioxidant	Mayonnaise	Mayonnaise was prepared with *P. atlantica* oil (PAO) with different substitution levels for soybean oil.	Introducing PAO to mayonnaise boosted its resistance to oxidation but decreased sensory scores and emulsion stability. Mayonnaise with 15% PAO was shown to have the best emulsion and sensory quality, while retaining its oxidative stability.	[87]
*Pistacia vera* L.	External hull	Antioxidant	Meat	*Pistacia vera external* hull was used to isolate polysaccharides, which were later added to minced meat.	Treatment reduced lipid oxidation during chilled storage and improved meat colour stability, in addition to exhibiting strong overall antioxidant properties.	[31]
*Pistacia atlantica* Desf.	Essential oil	Antimicrobial	-	A gelatine–carboxymethyl film was prepared with different levels of *P. atlantica* added.	Increasing the amount of essential oil significantly increased antimicrobial activity at the expense of the physical properties of the film.	[88]
*Pistacia terebinthus* L.	Fruit extract	Antimicrobial	-	Fruits hexane extract was obtained via a soxhlet system.	The extract showed strong antimicrobial effects against tested pathogens, especially *E. coli*, while exhibiting lower inhibitory properties against lactic acid bacteria, showing potential for use alongside probiotic strains.	[89]
*Pistacia khinjuk* Stocks	Fruit extract	Antioxidant	Sunflower oil	*P. khinjuk* fruit extract was obtained through the sonication method and used to prepare nanoemulsions with balango and fenugreek seed gum.	Oils stored for 24 days at 60 °C with the nanoemulsion showed improved stability and slower oxidation rates, though the peroxide, acid, and p-anisidine levels were increased.	[90]
*Pistacia atlantica* Desf.	Essential oil	Antifungal and antioxidant	Grape	Grapes were coated with a mixture of carboxymethyl cellulose and *P. atlantica* essential oil.	The treated grapes showed higher titratable acidity, antioxidant capacity, phenol, tannin, and anthocyanin contents, while also exhibiting delayed grape weight loss and fruit decay.	[91]
*Pistacia vera* L.	Resin and essential oil	Antimicrobial	Chicken breast fillets	Using wheat gluten, as well as *P. vera* tree resin and essential oil, an edible coating was created and applied to chicken breast fillets.	Samples with the coating applied exhibited significantly lower microbial growth and better sensory qualities (smell, texture, and appearance) compared to uncoated controls.	[92]
*Pistacia terebinthus* L.	Resin	Antimicrobial and probiotic preservation	Cheese whey beverages	*L. casei* probiotic cells were immobilised on *P. terebinthus* resin, which was then added to the cheese whey beverage.	Cheese whey treated with the resin did not present any spoilage microorganisms, while the probiotic cells present demonstrated high viability over a 30-day storage period at 4 °C. The product was also regarded to have a better aromatic profile.	[93]
*Pistacia* sp.	Seed hull extract	Antioxidant and antimicrobial	Chicken burger	Pistachio hull water extracts were prepared and used to treat chicken burgers.	In treated burgers, the cooking yield and moisture retention improved, the total phenolic content increased, and no significant differences in fat, ash, and protein contents were observed.	[94]
*Pistacia* sp.	Green hull extract	Antioxidant and antimicrobial	Beef patties	Lyophilised pistachio green hull water extracts were added to beef patties in different concentrations.	The addition of the extract at over 500 mg/kg has reduced lipid and protein oxidation and decreased metmyoglobin and discolouration. The antimicrobial effect was also observed against *S. aureus*, mould, yeast, and lactic acid bacteria, with no effect on *Enterobacteriaceae* noted.	[95]
*Pistacia lentiscus* L. var. *chia*	Essential oil of mastic gum	Antibacterial	- Tested on a model food system	Growth cultures of various bacteria were subjected to *P. lentiscus* essential oil of mastic gum.	The essential oil exhibited high antimicrobial properties, inhibiting the growth of bacteria such as *S. aureus* and *S. enteritidis*, with stronger effects against Gram-positive bacteria.	[96]
*Pistacia lentiscus* L.	Leaf	Antimicrobial and antioxidant	-	Essential oil was extracted from *P. lentiscus* leaves and subjected to broad analysis.	The extract was shown to have strong antioxidant and antimicrobial properties, as well as nutraceutical preserving effects.	[97]
*Pistacia terebinthus* L.	Extract	Antioxidant	-	A bilayer food package film was made using polylactic acid (PLA) and bitter vetch seed protein incorporated with *P. terebinthus* extract.	The addition of *P. terebinthus* extract to the film increased antioxidant capacity, flexibility, and thickness of the film, while decreasing moisture content, water solubility, and water vapour permeability. These desirable effects were observed at the cost of lower tensile strength and Young’s modulus.	[98]
*Pistacia* sp.	Green hull essential oil	Antifungal	Fresh in-hull pistachio	A nanocomposite was created using PVC, zinc nanoparticles, and pistachio green hull essential oil.	The essential oil rich in α-pinene and limonene showed strong antifungal effects, with high effectiveness against *Aspergillus flavus,* a fungus with large influence on the quality of pistachio. The composite proved suitable for maintaining quality of pistachios in a 60-day packaging evaluation.	[99]
*Pistacia atlantica* Desf.	Gum essential oil	Antimicrobial	Milk	A film based on polypropylene polymer, coated with silica nanoparticles and *P. atlantica* gum essential oil, was prepared.	The film was evaluated over a period of 35 days on bacteria including *E. coli* and *S. enterica.* It exhibited strong antibacterial properties, with the strongest effect on *S. enterica*. The incorporation of milk into the testing environment showed no significant effect on the antibacterial properties.	[100]
*Pistacia atlantica* Desf.	Essential oil	Antimicrobial	-	A film based on gelatine and carboxymethyl cellulose was prepared with the addition of *P. atlantica* extract.	With increasing essential oil content, the water vapour permeability, thickness, tensile strength, and solubility decreased, while antibacterial properties improved considerably.	[88]
*Pistacia vera* L.	Dehulling process waste	Antioxidant and antimicrobial	-	Chitosan films with different contents (2%, 4%, 8% *w*/*v*) of pistachio hull methanol extracts (PHEs) were prepared.	The chitosan-based films with 8% PHE showed high antimicrobial effects on all tested microorganisms. The 2% exhibited antioxidant properties, and the 4% PHE demonstrated the highest tensile strength and elongation at breakage.	[101]
*Pistacia terebinthus* L.	Stem, leaf, and seed extracts	Antioxidant and antimicrobial	-	Chitosan-based films were prepared with stem, leaf, and seed extracts of *P. terebinthus.*	With the incorporation of the extracts into the films, the antioxidant and antimicrobial properties improved, alongside increased biodegradability and elasticity.	[102]
*Pistacia vera* L.	Hull extract	Antifungal and antioxidant	-	Low-density polyethylene was used in the preparation of films with varying proportions of *P. vera* extract used as the active agent.	With the introduction of the extract, antifungal and antioxidant properties of the film were increased, with optimal release observed at 2 wt%. Heat resistance was also enhanced, but the tensile strength and elongation at break were lowered.	[103]
*Pistacia lentiscus* L.	Fruits and leaves	Antioxidant and antifungal	-	Packaging materials were prepared with *P. lentiscus* extract-based active adhesive between a layer of polyethylene terephthalate and a layer of low-density polyethylene.	The prepared extracts exhibited strong antioxidant and antimicrobial effects, with the leaf extract surpassing the properties of the fruit extract. The antioxidant capacity of the prepared film was also confirmed.	[104]
*Pistacia terebinthus* L.	Essential oil	Functional/ structural	Fresh cheese	Biocomposite films were prepared with soy protein and *Chondrus crispus* mucilage enriched with *P. terebinthus* essential oil (TEO).	Higher TEO concentrations were associated with thicker films. Lower concentrations (0.25% and 0.5%) resulted in greater elasticity, which decreased at higher concentrations. At 0.25% and 1% TEO, the oxygen barrier properties were found to be the most optimal. When tested on cheese, the films limited changes in pH, weight, and colour during storage.	[105]
*Pistacia lentiscus* L.	Leaf extract	Antimicrobial and antioxidant	Shrimp	Films were prepared with crosslinked carboxymethyl cellulose and gelatine as the base and Ge-montmorillonite, anthocyanins (ATH), and *P. lentiscus* extract (PE) as additives.	The incorporation of PE into the films significantly boosted their antioxidant and antimicrobial properties. The increase in PE concentration also reduced moisture content, swelling, and water vapour permeability and increased compactness and surface roughness, without affecting thermal stability. Films with the highest concentration of PE also performed best in shrimp spoilage tests.	[106]

**Table 3:** Corrosion-inhibitory performance of *Pistacia* species extracts on metallic surfaces.

Inhibitor Source	Metal/Alloy	Oil/Extract Type	Concentration	Medium	Isotherm Model	Highest Inhibition Efficiency	Effects and Methods	References
Galls from *Pistacia* Atlas	X-70 steel	Extracts obtained through Soxhlet extraction or maceration; solvents used: petroleum ether, dichloromethane, acetone, and methanol	84.3–2108.9 mg/L	1 N sulfuric acid	Frumkin	92.31% at 227 mg/L using methanolic extract obtained through Soxhlet extraction	The inhibition efficiency increases proportionally with the concentration of the extract and depends on the extraction solvent. The extract affected both anodic and cathodic reactions and performs well even at high temperature.	[110]
Galls from *Pistacia integerrima*	Mild steel	Aqueous extract achieved in Soxhlet apparatus	0–2000 mg/L	1 M H_2_SO_4_	Langmuir	92.19% at 2000 mg/L	Chemical constituents of gall extract, mostly pistciaphenyl ether, pistiphloroglucinyl ether, and naringenin, play key roles in its anticorrosive behaviour on steel in acidic medium. The inhibition efficiency increases proportionally with the concentration of the extract.	[109]
Leaves of *Pistacia lentiscus*	Mild steel	Oil and extract; hydrodistillation using a Clevenger-type apparatus	0.001–1 g/L	1 M HCl	Langmuir	Oil: 96.34% at 1 g/L Extract: 86.59% at 1 g/L	The inhibition efficiency increases with increased organic oil and extract concentration. Both acted predominantly as a mixed inhibitor type for the corrosion of steel in 1 M HCl without modifying the mechanism of hydrogen evolution reaction.	[111]
Galls from *Pistacia atlantica*	Mild steel X52	Ethyl acetate extract	0–25 ppm	1 M HCl	Langmuir	94.08% at 25 ppm	The extract showed excellent inhibition efficiency at 25 °C. The investigated galls-derived extract can be classified as a cathodic inhibitor. Increasing *Pistacia atlantica* extract concentrations were associated with higher Rct values and lower-capacitance Cdl.	[112]
Leaves of *Pistacia therebintus* L.	Iron	Methanolic extract obtained in Soxhlet apparatus	25–200 ppm	3% NaCl solution	-	96.96% at 200 ppm	The tested extract attenuated the cathodic process by influencing the corrosion mechanism. The inhibition efficiency increased proportionally with the concentration of the extract.	[113]
Twigs, leaves, and fruit of *Pistacia therebintus* L.	Iron	Essential oils obtained through hydrodistillation	1000–3000 ppm	3% NaCl medium	-	86.4% at 3000 ppm	Fruit EO (3000 ppm) demonstrated better anticorrosive protective properties than leaf and twig EOs. Also, a-Terpineol (447 Kcal/mol) had higher binding energy then other tested compounds.	[114]
Aerial parts	Copper and α-brass	Methnolic extract	50–300 ppm	Nitric acid solution (70% HNO_3_ with bidistilled water)	-	Copper: 91% at 300 ppm α-brass: 98% at 300 ppm	The tested extract is a good inhibitor; it acts as both a mixed-type and cathodic inhibitor for copper and brass corrosion in 1 M HNO_3_ solution. Inhibition efficiency increases proportionally with the concentration of the extract.	[115]

**Table 4:** Potential cosmeceutical and dermatoprotective roles of *Pistacia* sp. extracts and derivatives.

Plant Source and Form	Target Effect	Activity	Active Ingredients/Indicated Compounds	Form/Delivery Route	Formulation Type	Effects and Methods	References
Resin from *Pistacia lentiscus*	Anti-pruritic, allergic dermatitis treatment	Modulating keratinocyte activation in a mouse model of allergic dermatitis	-	Topical treatment	Highly purified mastic; dissolved in caprylic/capric triglycerides to prepare a final concentration of 1%, 3%, 5%, or 30%.	Topical treatment with mastic significantly ameliorated ear swelling, itch behaviour, immunocyte infiltration, and cytokine production. The anti-inflammatory responses were confirmed by histological evaluation.	[119]
*Pistacia atlantica* resin (essential oil and methanolic extract)	Anti-adherence and antibacterial activities	Reduction in *Streptococcus mutans* from the oral cavity or its adherence to tooth surfaces.	-	300 mg/mL of both extracts in DMSO; diluted extracts at concentration 60–100% were added to each well of 96-well microtiter plates with the *S. mutans* suspension		The highest anti-adherence activity (about 81%) was observed for 100% EO, while 100% ME exhibited the lowest anti-adherence activity (22.9%). These results suggest that *P. atlantica* may have potential anti-adherent activity and could be of value in oral care.	[120]
Vegetable oil extracted from *Pistacia lentiscus*	Natural alternative for synthetic active ingredients in antifungal agents	Antifungal and antibacterial activity (against *Staphyloccocus aureus*, *Pseudomonas aeruginosa*, *Escherichia coli*, *Candidas albicans*); antioxidant (anti-radical) activity	Free fatty acids (oleic acid, palmitic acid, or auric acid); phenolic compounds, flavonoids (quercetin)	Emulsion	Antifungal emulsion based on *P. lentiscus* oil extracted at different percentages (0.5, 1.5 and 2%).	IC_50_ values for extracts from Tizi ouzou and Boumerdes oils were respectively 919.405 mg/mL and 948.06 mg/mL. The tested emulsions prevented fungal and bacterial development. The emulsions were found to be moisturising, homogeneous, and easy to apply and incorporate into the skin. There were no side effects such as skin allergies.	[121]
*Pistacia atlantica* gum	Topical patch adhesive agent	Antifungal in patches as drug carriers; the use of *P. atlantica* gum as an adhesive agent	Not applicable	Drug-in-adhesive patch containing an active ingredient as a possible method for developing an antifungal nail patch with desired properties through the development of a systematic approach		The *P. atlantica* gum demonstrated adhesion properties like tack and peel, 0.32 ± 0.03 N/mm^2^ and 5.34 ± 0.52 N/25 mm, respectively. The film also exhibited high mechanical properties (elongation at breakage and Mpa modulus). The antifungal activity of *Pistacia* was not tested, although its gum may be a great natural substitute for synthetic polymers in patch production.	[122]
*Pistacia atlantica var. mutica* gum	Oral and gingival care	Anti-halitosis and antimicrobial activity	Alpha-pinene, beta-pinene, sabinene, myrcene, limonene, camphenol, trans-verbenol	Toothpaste	Freeze-dried tested ethanolic solution was diluted 5-fold with standard toothpaste vehicle.	The organoleptic properties, phase separation, particle size, and microbial tests of formulations showed an accepted shelf life for performing clinical trials.	[123]
Aerial parts of *Pistacia atlantica var. mutica*			-	Mouthwash	To prepare the mouthwash solution, the essential oils were dissolved in a mixture of water and alcohol (80:20) at 1.2%.	*P. atlantica* mouthwash was better tolerated and caused fewer side effects than control mouthwashes. It may be used as an effective alternative against gingival microorganisms.	[117]
*Pistacia vera* by-products	Cosmetics formulation	Emulsion properties; foaming properties; antioxidant activity	Protein, carbohydrates, fat	Dry samples treated with six volumes of 95% ethanol, and the dried residue was extracted twice with deionized water; it was then combined and filtered, evaporated, precipitated in alcohol, and re-dissolved in distilled water; dialyzed and concentrated to obtain polysaccharides		Pistachio juice polysaccharides had good water-holding ability, fat-binding capacity, foaming properties, and emulsification stability. Furthermore, the results showed that polysaccharides of pistachio juice were effective antioxidants in vitro and in a dose-dependent manner.	[124]
*Pistacia lentiscus* L. extract	Hair growth promotion	Human dermal follicle papilla cell proliferation; anti-inflammatory and antioxidant effect	-	Topical	Mastic gum extract prepared from mastic gum powder and ethanol was combined with peppermint extract in ratios of 7:3.	Mastic gum greatly supported cell proliferation and demonstrated synergistic elevation of activity when combined with peppermint extract.	[125]
*Pistacia atlantica* fruit-derived oil-encapsulated chitosan nanoparticles	Skin care applications	Biocompatibility; maintaining viability of cells; antioxidant activity; wound healing	Phenolic compounds, flavonoids, fatty acids (oleic acid, linoleic acid, etc.), protein, carbohydrates	Nanoparticles, nanocomposites, nanofibers	*P. atlantica* oil was encapsulated within chitosan nanoparticles through ionic gelation; nanoparticles were embedded into PVA nanofibers	Antioxidant assays showed robust activity with FRAP and DPPH values of 79.4 µmol ESF/100 g and an IC_50_ of 7344.7 µg/mL. The incorporation of *P. atlantica* oil with nanoparticle system supported controlled release and biocompatibility.	[126]
Galls of *Pistacia integerrima* Stew ex Brandis	Anti-leishmanial effect	Leishmanicidal potential; arginase inhibition	Flavonoids	Extract	Isolation of flavonoids through extraction with hexane and chloroform/ethyl acetate; subjected to thin layer chromatography and column chromatography	Both isolated flavonoids exerted significant leishmanicidal activity, making them a highly attractive potential drug for leishmaniasis treatment. The two isolated compounds docked well into the active site of arginase and formed strong hydrogen bond interactions with the receptor.	[127]
*Pistacia lentiscus* gum	Nail strengthening	Improvement in nail appearance and weakness; increase in viscoelasticity and firmness; increase in the smoothness of the surface	-	Water-soluble nail strengthener (WSNS)	The active product was obtained through combination of silanediol salicylate and *Pistacia lentiscus* gum. The product also contained hyaluronic acid.	The participants reported that the tested WSNS was well tolerated and resulted in less brittleness and better appearance after 84 days. *In vitro* study found the WSNS to increase nail firmness.	[128]
						After six months of treatment, 76% of patients reported better nail appearance, greater nail plate roughness, higher nail resistance, and reduced breakage. All patients reported an improvement after 1, 3, and 6 months.	[129]
*Pistacia lentiscus* var. *chia* gum	Cutaneous application	Antimicrobial and anti-inflammatory properties	-	Polymeric nanoparticles (NPs)	Solvent evaporation method/single emulsion technique; nanoparticles were prepared utilising poly(lactic acid) (PLA) as shell material and poly(vinyl alcohol) (PVA) or lecithin (LEC) as surfactants.	The PLA/PVA-NPs demonstrated an efficient nanoencapsulating structure and better release sustainability than PLA/LEC-NPs. The NPs showed low/none antimicrobial activity, suggesting no harmful effect on skin microbiota.	[130]
*Pistacia vera* L., *variety Bronte* seed (SP) and skin (TP)	UV-B-induced skin erythema treatment	Antioxidant and photoprotective effect	Phenolic acids, flavan-3-ols, isoflavones, flavanones, flavones	SP extract	Decorticated seed powder was extracted with hexane through homogenization and ultrasonication (three times).	Both extracts demonstrate good antioxidant (IC_50_ values were 0.26 ± 0.02 mg/mL and 2.06 ± 0.18 mg/mL, for TP extract and SP extract respectively) and photoprotective activity (2.49 ± 0.18 mg/mL and 4.05 ± 0.36 mg/mL for TP extract and SP extract respectively).	[131]
			Phenolic acids, anthocyanins, catechin, epicatechin, flavanones, flavonols, flavones	TP extract	Methanol/water mixture was used for the extraction from crushed skin; treated with homogenization and ultrasonication 3 times.		
*Pistacia terebinthus*	Soap in skin toxicity treatment	Treatment of cetuximab-induced skin lesions and erythema	-	Soap (the “bittim” soap); topical administration	-	Among the patients treated with the soap, complete response was noted by 100% of those with grade 2 skin toxicities and 33% with grade 3. In the remaining patients with grade 3 skin toxicity, the toxicity regressed to grade 1. Skin toxicity reoccurred in all patients after stopping administration; it was therefore used throughout the cetuximab treatment.	[132]
*Pistacia atlantica*	Cutaneous wound healing	Antioxidant activity; morphological correction of wound formation; re-epithelialization; reduction in neutrophils and lymphocyte; and increase in fibroblasts	-	Topical application of *P. atlantica* gel	The fruit powder was extracted with n-hexane by mixing in a dark place at ambient temperature for 48 h. The extract was incorporated into the gel base made of carbopol, sodium hydrochloride, and distilled water.	Topical application of the tested gel in rats improved the biomechanical properties of induced wound defects. The tested gel improved re-epithelialization with continuous stratum basalis and a mature granulation tissue and adnexa and organised the collagen fibres.	[133]
*Pistacia lentiscus* fruits	Burn wound healing	Promotion of wound contraction and epithelialization acceleration	Fatty acids (palmitic 16.3%, oleic 55.3%, and linoleic 17.6%)	Virgin fatty oil	Fruit was air dried in shade and the oil was extracted through cold-pressing.	*Pistacia lentiscus* virgin fatty oil promotes wound contraction and reduces epithelization period in rabbit model significantly more efficiently than commercially used wound healing ointments.	[134]
*Pistacia lenticus* fruit	Wound healing	Cream preparation for healing wounds after laser skin resurfacing	-	Topical administration of oil-in-water emulsion	Oil-in-water emulsions with different internal phase concentrations of *Pistacia lentiscus* fruit oil (5, 10, and 20%).	Increasing concentrations of *P. lentiscus* fruit oil demonstrated higher viscosity, stability, and viscoelasticity. However, high fruit oil levels demonstrated non-homogenous distribution of the droplet size, suggesting a dose-dependent activity. Fruit oil may be of value in wound healing cream production.	[135]
*Pistacia* sp. hull	Wound healing	Anti-inflammatory, antimicrobial, and antioxidant effects.	-	Ointment for topical use	Hydroalcoholic extract ointment 1%, 2%, and 4%.	The incorporation of pistachio hull extract increased the thickness of skin and collagen diameter, reduced oedema, and decreased the inflammatory cell count significantly; the extract demonstrated great anti-inflammatory effects, supporting wound healing.	[136]
*Pistacia lentiscus* L.	Anti-ageing	Antioxidant; tyrosinase, elastase, and hyaluronidase inhibition	Galloyl derivatives (quinic acids and catechins); flavonol derivatives (quercetin, kaempferol, and myricetin)	-	Aqueous extract obtained from leaf hydrodistillation residues.	The tested extract exhibited an inhibitory effect on skin-ageing enzymes with IC_50_ = 33.8 µg/mL (tyrosinase), 17.4 µg/mL (elastase), and 4.3 µg/mL (hyaluronidase), while showing no collagenase inhibition. These findings, along with high antioxidant effect, suggest a great anti-ageing potential.	[137]

**Table 5:** Potential utilisation of *Pistacia* sp. in the development of functional materials.

*Pistacia* Species	Type of Material	Part of Plant	Obtaining Method	Effects	Reference
*Pistacia vera* L.	Polymer composites	Shell	Mixtures of pistachio shell powder at different proportions and volumes were added to the composite. The samples were shaped and cured using mechanical mixers and silicone moulds.	The composite with 30% volume of pistachio shell powder and AAR resin demonstrated 15.37% better flexural strength, 22.7% better modulus, and 18.4% greater impact strength compared to plain epoxy resin, as well as 30% improved damping properties. It also demonstrated improved biodegradability, with a 5.2% weight loss in 120 days when buried in wet soil.	[142]
*Pistacia atlantica Desf.* subsp. *mutica (Fisch. & C.A.Mey.) Rech.f.*	Edible biopolymers	Resin	Poly(lactic acid) (PLA) and *Pistacia atlantica* subsp. mutica gum (PAG) were mixed in different proportions and treated with a plasticiser and a reactive compatibiliser to prepare sample sheets.	The 70/30 (PLA/PAG) ratio was found to exhibit the best mechanical properties, with the greatest elongation at break (improved flexibility), the lowest yield strength and Young’s modulus (reduced stiffness), as well as the best moisture resistance and the highest polymer chain mobility. The material was also found promisingly biodegradable, with degradation exceeding 50% after 6 months.	[143]
*Pistacia lentiscus* L.	Nitrogen-doped nanoporous bio-graphene	Resin	*Pistacia lentiscus* gum was used as a source of nitrogen rich natural carbon and the bio-template for the synthesis of the material.	The usage of *P. lentiscus* in the synthesis of the material improved its adsorption properties by increasing porosity and providing nitrogen functional groups, improving the overall affinity for polar compounds.	[144]
*Pistacia* sp.	Anode materials	Shell	Pistachio shells were used to create hard carbon materials for use in sodium-ion batteries by hydrothermal treatment and carbonisation.	The electrode with hard carbon prepared at 1000 °C exhibited the best properties out of the studied samples, with the best storage performance and high reversible capacity and good long-term performance (even after 500 charges).	[145]
*Pistacia* sp.	Rubber	Shell	Biochar made from pistachio shells were used as a substitute for carbon black in the preparation of rubber.	Up to 40% of carbon black could successfully be replaced in the process; the biochar demonstrated better tensile strength and toughness, though with some reduction in modulus.	[146]
*Pistacia* sp.	Biodegradable films	Shell	Hemicellulose from pistachio shells was incorporated into biodegradable films of gelatine and glycerol.	The optimal properties were observed for the film with a hemicellulose/gelatine ratio of 35.93% and glycerol ratio of 18.02%; at this value, the film demonstrated 4.34 times greater elongation at break and improved water permeability and biodegradation compared to conventional gelatine films. The tensile strength of the sample and its water solubility were, however, decreased.	[147]
*Pistacia vera* L.	Bio-filler for eco-friendly composites	Shell	*Pistacia vera* shells were cleaned, dried and ground to create fine powder to prepare for testing.	Powdered *Pistacia vera* shell was found to be a good eco-friendly filler, with a suitable structure (porosity and surface roughness) and thermal stability under 240 °C without prior treatment.	[148]
*Pistacia* sp.,	Asphalt	Shell	Samples were prepared incorporating the pistachio shell powder to an asphalt mixture at an elevated temperature and with continuous mixing for homogeneity.	When added to the material in concentrations up to 6 wt%, pistachio shells acted as a thermal stabiliser increasing the degradation temperature.	[149]
*Pistacia* sp.	Cellulose-based nanocomposites	Shell	Cellulose was extracted from pistachio shells through chemical treatment and was used for the preparation of nanocomposites.	The extraction processes employed were effective in obtaining 95% crystalline cellulose, which, when incorporated into a matrix material, significantly increased tensile strength, tensile modulus, as well as the flexural strength and modulus.	[150]
*Pistacia vera* L.	Cellulose nanocrystals	Shell	To extract cellulose, cleaned and ground pistachio shells were purified, after which they were subjected to hydrolysis to obtain nanocrystals.	The obtained nanocrystals were found to have comparable crystallinity and surface charge density to commercially available, wood-sourced alternatives, while possessing a yield of around 50%—high for an agricultural waste product.	[151]
*Pistacia* sp.	Cement	Shell	Pistachio shells were burnt to ash and mixed with commercially available cement to prepare test specimens.	The addition of pistachio shell ash up to 20% by weight yielded similar or higher compressive strength, as well as improved microstructural and mechanical properties, compared to conventionally prepared cement.	[152]
*Pistacia* sp.	Polypropylene	Shell	Samples of polypropylene, both pure and with the addition of pistachio shell powder (among other nuts), were prepared for testing using microcompounding and injection moulding.	The addition of pistachio shell powder at certain proportions significantly improved tensile strength, sliding wear resistance, and stiffness, though at the cost of the material becoming more brittle compared to conventional polypropylene. Pistachio shell powder was also the most effective at enhancing scratch resistance out of the tested nutshell powders.	[153]
*Pistacia vera* L.	Poly(butylene succinate) composites	Shell	Pistachio shell flour (PSF) was used as filler in the preparation of poly(butylene succinate) composites, with a grafted compatibiliser (PBS-g-MAH) and melt extrusion followed by injection moulding.	The resulting composites demonstrated a wood-like colour and significantly greater mechanical and thermomechanical rigidity and hardness; this was attributed to a more crystalline structure, good filler dispersion, and improved matrix–filler interface interactions compared to poly(butylene succinate) without PSF or the compatibiliser.	[154]
*Pistacia* sp.	Polypropylene	Shell	Polypropylene matrix composites were prepared with the usage of chemically treated pistachio shells as filler.	The addition of pistachio shell particles improved crystallinity, thermal stability, and biodegradability compared to conventional polypropylene.	[155]
*Pistacia lentiscus* L.	Microencapsulating and matrix-forming material	Resin	Mastic, the natural resin from the *P. lentiscus* tree, was used to prepare microparticles using the oil-in-oil solvent evaporation method. Matrix tablets were obtained by wet and melt granulation, with diclofenac sodium (DFS) and diltiazem hydrochloride (DLTZ) as model drugs.	Mastic formed microspheres with DLTZ and microcapsules with DFS, with the increase in resin content, decreasing the drug release rate, improving drug loading, and increasing microparticle size. The results showed that mastic can be used to prepare tablets with properties acceptable for pharmaceutical applications.	[140]
*Pistacia* sp.	Ceramic bricks	Shell	Pistachio shells were used as a pore-forming agent in the production of ceramic bricks. Samples with different biomass proportions were shaped appropriately and heat treated.	The best properties were noted for 10% of added biomass, with comparable flexural strength and porosity to commercially available products. At higher percentages of added pistachio shells (15 and 20%), the material showed lower overall mechanical properties, most likely due to the formation of pore agglomerates in the structure.	[156]
*Pistacia* sp.	Glass fibre polymer composites	Shell	The vacuum assisted resin infusion moulding (VARIM) method was used to prepare composite laminates from chopped strand mat glass fibre and different weight percentages of pistachio shell particles.	The tensile strength increased by 54.05% for 3 wt% pistachio filler; the flexural strength increased by 22% for 2 wt% filler. The improvements were attributed to uniform dispersion of filler, as well as good fibre–matrix bonding.	[157]
*Pistacia* sp.	Polymer composite	Shell	Specimens were prepared by the hand lay-up technique, with pistachio shells used along with a hardener and an unsaturated polyester to create the composite.	The greatest impact strength and flexural strength were noted for 5 wt% pistachio shell particle content; the properties decreased as wt% increased.	[158]
*Pistacia* sp.	Epoxy composites	Shell	Pistachio shell powder was used as bio-filler for preparation of a composite from a commercially available epoxy resin and a hardener used as matrix materials. To prepare the specimens, the substances were mixed, vacuumed and left to cure at room temperature.	Pistachio powder increased maximum bending and tensile strength by 15% and 8%, respectively. Hardness increased by 8% and the density value by 1.7%, with no air bubbles or agglomeration detected in the structure.	[159]
*Pistacia lentiscus* L.	Bio-silver nanoparticles on cellulosic fabrics	Peel	*P. lentiscus* peel extract was prepared, mixed with silver nanoparticles and applied to a cotton fabric with the usage of a microwave-assisted method.	Pistachio peel extract augmented the properties of the cotton fabric. The material exhibited high durability against friction and light exposure, good colour stability, as well as good antibacterial performance, with 100% inhibition of *S. aureus* and *E. coli.*	[160]
*Pistacia* sp.	Polymer matrix composite	Shell	A composite particleboard was created from crushed pistachio shells alongside fly ash.	The material exhibited an increase in hardness and three-point bending strength with the increase in the urea-formaldehyde/pistachio shell ratio, with the best properties achieved at the ratio of 1 (*w*/*w*), after which the properties started decreasing slightly.	[161]
*Pistacia vera* L.	Graphene oxide/carbon quantum dots	Shell	Hard pistachio shells were used as the carbon nanomaterial preparation source, which was subjected to milling and hydrothermal synthesis and treated with other substances.	Two composites were detected in the final product: spherical structures with a core synthesised from Fe_2_O_3_ surrounded by a shell of carbon dots, as well as two-dimensional graphene oxide sheets. The obtained substances were found to be suitable for photocatalytic dye degradation and heavy metal adsorption.	[162]
*Pistacia vera* L.	Carrageenan-based composite	Shell	Pistachio shells were used to isolate microcrystalline cellulose, which was later used as filler in carrageenan films.	The carrageenan films synthesised with the added microcrystalline cellulose exhibited better thermal stability, mechanical properties, higher UV resistance, and improved oxygen barrier properties.	[163]
*Pistacia* sp.	Microporous carbon for supercapacitors	Shell	Pistachio shell biomass was pyrolysed and activated using KOH to prepare microporous carbon.	The synthesised material showcased high porosity and a large surface area, together with good electrochemical properties, large areal capacitance, and significant energy capacity.	[164]
*Pistacia atlantica* Desf.	Nanofibre–hydrogel	Resin	Nanofibres composed of polyamide and *P. atlantica* gum were produced via electrospinning and applied as a biological layer on a PEBAX/PVA hydrogel embedded with silver nanoparticles, intended for wound dressing applications.	The addition of *P. atlantica* gum augmented mechanical properties of the material—particularly tensile strength—while the biological nanofibre layer improved the hydrogel’s wound healing performance, including water absorption, vapour permeability, and antibacterial activity.	[165]
*Pistacia atlantica* Desf.	Nanofibres	Oil	Polyvinyl alcohol (PVA)-sodium alginate (SA) nanofibres loaded with *P. atlantica* oil (PAO) were produced using the electrospinning technique.	The incorporation of PAO increased nanofibre diameter and improved scaffold stiffness, with the highest value at 1.5% *w*/*v* PAO concentration, along with enhanced thermal stability and crystallinity. The optimal, controlled, and efficient drug release was reached at 1.5% *w*/*v* PAO concentration.	[141]
*Pistacia atlantica* Desf.	Silver, zinc nanoparticles, and silver–zinc oxide nanocomposites	Resin	The resin extract of *P. atlantica* was used as a reductant and capping agent in the nanocomposite and nanoparticle biosynthesis process.	Synthesis was successful and resulted in greater antibacterial properties. It also replaced the chemical reducing and stabilising agents, making the synthesis more environmentally friendly.	[166]
*Pistacia* sp.	Microporous carbons	Shells	A CO_2_ activation process has been used on pistachio shell biomass pretreated in different ways to create microporous carbons.	Pistachio shell biomass was found suitable for the synthesis of highly nanoporous carbons with the usage of hydrothermal carbonisation prior to CO_2_ activation, with potential for tailoring the pore structure depending on the pretreatment method used.	[167]
*Pistacia vera* L.	Nanocomposite packaging	Hull	*P. vera* hull essential oil and zinc nanoparticles were used to produce a packaging film, with PVC functioning as the matrix.	The synthesised material was found to extend shelf life, with the essential oil possessing antifungal properties, especially against *Aspergillus flavus*, due to its high content of monoterpenes.	[99]
*Pistacia* sp.	Pellets	Pruning residues	The residues, including leaves and stems, left behind by the pruning of pistachio trees were ground and pressed to produce the pellets.	With proper optimisation, pistachio tree pruning waste was turned into pellets with desirable density, strength, and durability; the best properties were achieved at 100 °C pelletising temperature, 1.65 mm particle size, and 11.7% moisture content.	[168]
*Pistacia* sp.	PLA biocomposites	Shell	Ground pistachio shells were used in the preparation of a poly(lactic acid) (PLA) composite with alkaline–silane pretreatment applied to enhance interfacial adhesion.	With 20% of added pretreated pistachio shells, the material exhibited elevated flexural modulus and tensile strength (18.63% and 46.9% increase, respectively), alongside smaller susceptibility to thermal decomposition.	[169]
*Pistacia* sp.	Unsaturated polyester matrix composite	Shell	Pistachio shell particles (PSPs) were incorporated into a polyester resin composite at 10, 20, 30, and 40 wt% via hand mixing and compression moulding.	Maximum tensile strength and flexural strength are found at 40 wt% of the added PSP, with the highest impact strength at 10 wt% PSP and thermal stability increasing with the increase in PSP content.	[170]
*Pistacia vera* L.	Bio-based composite films	Shell	*Pistacia vera* shells were used, alongside other materials, for the production of biocomposite films, with the use of different plasticising, thickening, and stabilising agents.	The biocomposite demonstrated reduced water solubility, moisture content, and swelling index compared to polyethylene material, as well as higher biodegradability. However, the product was characterised by higher vapour transmission and worse antimicrobial properties.	[171]
*Pistacia* sp.	Natural rubber/styrene–butadiene rubber-based rubber compounds	Shell	Ground pistachio shells were used as filler in the production of rubber conveyor belt compound, as a material to partially replace carbon black in the formulation, achieved by the two-roll mill method.	The increase in ground pistachio shell content significantly improved the abrasion resistance of the materials at the cost of lowered cure extend and tensile strength.	[172]
*Pistacia* sp.	Bitumen and asphalt mixtures	Hard skin (endocarp)	Pistachio hard-skin ash (PHSA) was used in the preparation of asphalt mixtures at different proportions.	With the increase in PHSA content, the softening point and density of the mixtures were improved, together with better Marshall stability. However, dynamic shear modulus, penetration value, and ductility were reduced.	[173]
*Pistacia vera* L.	Thermoset resins	Shell	Pistachio shell powder was mixed with unsaturated polyester and vinyl ester resins, and the mixtures were cured with a cobalt-based accelerator.	Added pistachio shell filler absorbed UV light, worked as a charring agent, caused a plasticising effect in the polymer matrix, and increased water sorption.	[139]
*Pistacia* sp.	Sound-absorbing materials	Shell	Three different forms of pistachio shells were used in the preparation of samples: whole, semi-crushed, and crushed. The material was initially washed, dried, treated with boric acid, and finally ground into the appropriate form.	The samples exhibited good absorbance capabilities; these improved with layer thickness, density, and size of post-crushing debris. This increase also caused a shift in the absorption peak towards lower frequencies.	[174]

**Table 6:** Potential forms of application of *Pistacia* sp. in degradation and adsorptive removal of environmental and chemical pollutants.

Pollutant	Form and Species of *Pistacia*	Method of Preparation	Optimal Experimental Conditions	Efficiency and Adsorption Capacity	Adsorption	Note		Reference
Acid blue 113	*Pistacia atlantica Kurdica* nutshell	Nutshells were ground and sieved, purified from water-soluble compounds, dried, and rinsed with H_3_PO_4_. After decantation the material was subjected to 25–350 °C in He and NH_3_ atmosphere and it was microwaved at the end.	BMM-AC: 20 min, 0.82 g/L of the adsorbent, pH 7.25 AC: 45 min, 1.37 g/L, pH 4.35 BM-AC: 30 min, 1.18 g/L, pH 6.6. MM-AC: 1.18 g/L, pH 5.42	Basic and microwave- modified AC (BMM-AC): 97.54% AC: 60.32% Microwave-modified AC (MM-AC): 73.08% Basic modified AC (BM-AC): 88.72%	Freundlich model; chemisorption and physisorption	The best results for adsorption of acid blue 113 were demonstrated by basic and microwave modification of *P. atlantica* nutshell, suggesting great potential for acidic azo dye adsorption.		[182]
Acid red 183 (AR) Acid green 25 (AG)	*Pistacia khinjuk* Stocks shells	Washed, dried, crushed, and sieved.	At 45 min in 308 K: AR adsorption: 4 g of the adsorbent AG adsorption: 3–4 g of the adsorbent—pro rata with the initial concentration of the dye	AR: 29.7–68.8% depending on the initial concentration of AR AG: 11.2–32.4% depending on the initial concentration of AG	Langmuir model	The tested shell-derived biomaterial, primarily and agro-based waste, was tested for acid dye removal potential from aqueous solution. The results confirmed it can be used as a low-cost and zero-waste adsorbent.		[183]
Acid violet 17	*Pistacia* sp. shells	Washed, dried; PNS1, PNS2, and PNS3 were prepared by mixing nutshells with 2 parts of 18 N sulphuric acid and kept in furnace at room temperature, 333 K, and 353 K respectively. Washed.	pH of 2	Adsorption capacity Qm (mg/g): 125.00	Langmuir model	Tested pistachio nutshell was proved to be effective for acid violet adsorption, suggesting potential use in anionic dyes removal. Increase in temperature was to the advantage of adsorption capacity.		[184]
Azo dyes (Reactive red 238)	*Pistacia* sp. shells	Washed, dried, grounded, and sieved; the particles in the range of 63–125 μm were chosen for the study.	Adsorption reached the plateau at 10 min.	Maximum biosorption capacity of 109.535 mg/g	Sips model	The tested Pistacia spp. shells were very effective at adsorbing hetero-bireactive azo dyes from liquid solutions, although the efficiency decreased with an increase in dye concentration.		[185]
Antibiotics (trimethoprim, sulfamethoxazole, and sulfapyridine antibiotics)	*Pistacia vera* shell-derived biochars	Heated under N2 flow from ambient temperature to 400, 600, or 800 °C; pyrolyzed and cooled to room temperature; ground and sieved to 180 μm,	Increase in temperature decreases the level of adsorption of tested antibiotics.	Maximum removal efficiency for *P. vera* shell biochars TMP: 96.58% SMX: 98.1% SPY: 99.45%	Langmuir model	Antibiotic adsorption capacity generally decreased with increasing pyrolysis temperature; adsorption affinities for trimethoprim were temperature-independent, while affinities for sulfamethoxazole and sulfapyridine increased with pyrolysis temperature.		[186]
Basic blue 41 (BB 41)	*Pistacia* sp. shells	Washed, dried at 103–105 °C; pulverised, sieved.	pH of 9; 323 K for above 100 mg/L BB 41	Maximum adsorption capacity: 41.77 mg/g in continuous model Maximum observed removal: 95.54%	Langmuir model	Increasing pH and temperature supported removal efficiency and adsorption capacity of basic blue 41, suggesting its potential use in monoazobasic dye removal and adsorption in wastewater treatment.		[187]
Basic fuchsin	Raw *Pistacia* sp. nutshells	Seven adsorbents were developed; raw shells (RPNSs) and the thermally activated biomasses at six different temperatures (250–500 °C).	pH of 12, 100 mg/50 mL of RPNS, and 250 ppm BF for 20 min.	Removal: 99.71% Adsorption capacity: 118.2	Dubinin–Radushkevich (DR) and Langmuir isotherms	Raw shells turned out to be more effective than thermally activated biomasses derived from the same material.		[188]
Brillant Blue (BB), K-RED 198, Methylene Orange (MO), Methylene Blue (MB)	*Pistacia* sp. shells	The shells were cleaned with deionized water, dried and grinded by 1–1.7 mm intervals	pH of 7	Removal capacity BB: 65% KRED 198: 73% Adsorption capacity BB: 4.04 mg/g KRED 198: 4.64 mg/g	Freundlich Isotherm model	The dye removal activity of *Pistacia* shell was significantly higher for brilliant blue and K-RED 198 than for methylene orange and methylene blue.		[189]
Cibacron Blue (CB)	*Pistacia* sp. shells	Washed with deionized water, dried in ambient air, crushed, grounded, and sieved into max. 1.5 mm particles. Modified with aqueous HCl solution and with NaOH solution.	pH 2–3; adsorbent dose 10.00 g/L Unmodified powder: 1–12 h of contact time Modified powder: 2–12 h of contact time	Maximum adsorption capacity PSP: 1.63 mg/g TPSP: 4.53 mg/g	Langmuir isotherm	Treatment of pistachio shell powder with HCl and NaOH enhances the adsorptive potential for CB removal. The amount of dye adsorbed per unit mass of adsorbent decreased with increasing adsorbent mass.		[190]
Crystal violet (CV)	*Pistacia* sp. shells	Washed with distilled water, dried in the oven at 110 °C for 24 h. Crushed to the size of 1–2 mm and carbonised at 600 °C. Carbonised samples were washed and activated at 750 °C for 4 h.	pH of 7.5 Initial concentration of CV: 20 mg/L Adsorbent dose: 0.0088 Contact time: 10 min	Removal efficiency of 97.6% at optimal conditions	Langmuir monolayer isotherm model	Use of pistachio shells in preparation of activated carbon (pistachio/nanodiopside nanocomposite) may be potentially an efficient, environmentally friendly, and low-cost crystal violet removal method.		[191]
Direct blue 71	*Pistacia* sp. hull	*Pistacia* sp. hull was grounded and sieved to 20–100 μm mesh particles; washed using distilled water and dried at room temperature for 24 h and then dried at 85 °C for 72 h.	pH: 2 DB71: 100 mg/L Temperature: 50 °C Time: 210 min	Maximum adsorption capacity: 90.48 mg	Freundlich isotherm and Langmuir isotherm	*Pistacia* hull exerted good direct blue 71 removal properties depending on *inter alia* pH, initial dye concentration, contact time, temperature, and the amount of the adsorbent.		[192]
Malachite green oxalate (MGO), methylene blue (MB)	*Pistacia terebinthus* “coffee” waste	Bio-waste was mixed in deionised water, filtered, and stirred in an alcohol–water mixture; filtered again under vacuum and dried at 107 °C.	For 200 mg/L of dye solution at 25 °C for 45 min with a mixing speed of 400 rpm: MGO pH of 8, 150 mg of adsorbent MB pH of 6, 200 mg of adsorbent	Maximum dye adsorption 99.59 ± 0.05% for MGO 96.54 ± 0.21% for MB	Langmuir isotherm	*Pistacia terebinthus* (Menengic) coffee waste was used as a low-cost, eco-friendly adsorbent to remove MGO and MB dyes from water. It achieved up to 99.59% removal efficiency, showing high adsorption capacity and exothermic behaviour.		[193]
Methyl red, iodine, NO_2_/H_2_S gases	Pistachio nutshells	Cleaned, dried; subjected to carbonization; physically and chemically activated at different temperatures with eight types of samples: PC5, PC7, PC5PA, PC7PA, PD75, PD8, PAcH, and PAcK.	At pH of 5.4 Highest adsorption level NO_2_: 77.4 mg/g with PC7PA in humidity H_2_S: 46.4 mg/g with PC7Pa in humidity and 11.3 mg/g with PD8 under dry conditions Methyl red: 264 mg/g (PAcK) and 262 mg/g (PAcH) Iodine: 1281 mg/g (PAcK) and 11 mg/g (PC7PA)		Low-temperature nitrogen adsorption/ desorption isotherms	These activated carbons were found to show high effectiveness in removal of NO_2_ and H_2_S from the air stream and in removal of inorganic and organic pollutants from water.		[194]
Plant protection products: oxyfluorfen (OXY), metribuzin (MET), imidacloprid (IMI), and xenoestrogen bisphenol A (BPA)	Pistachio shells	Washed, soaked in distilled water, dried at 40 °C for 36 h, and ground to <1 mm particles.	24 h of contact pH of 4.95 ± 0.11	Highest adsorption capacity OXY: 713 mg*kg^−1^ MET: 317 mg*kg^−1^ IMI: 359 mg*kg^−1^ BPA: 736 mg*kg^−1^	Freundlich isotherm model	This study concludes that pistachio shells can behave as bioadsorbents of organic pollutants, especially highly hydrophobic ones. This is due to their large surface area and porosity, the abundance of reactive functional groups, and their composition. They can be used as low-cost adsorbent, which can be incorporated into the soil for enrichment in organic matter.		[195]
Reactive red 120 (RR120)	*Pistacia khinjuk* Stocks leaf extract	Processed in order to obtain biomass, biochar (collected through pyrolysis), and nanocomposite (ZnTiO_3_).	Leaves: washed, dried (72 h), and powdered Biochar: pyrolysis at 550 °C (2 h, limited O_2_) Nanocomposite: green synthesis using leaf extract + ZnSO_4_ + Ti precursor pH: 9–10 Calcined at 500 °C	Max degradation: 94% (ZnTiO_3_) Biochar: ~54% Biomass: ~42% Equilibrium reached ~120 min	Kinetics: pseudo-second order; isotherm model: not applicable-degradation	The highest photocatalytic potential for dye degradation was detected in the TiO_3_ nanocomposite model, whose performance is supported by high surface area and reactive oxygen species generation. Biochar was moderately effective due to its porosity, whereas biomass exerted the lowest efficiency.		[196]
Reactive red 120	Pistachio husk	Washed with distilled water, dried at 80 °C for 24 h; ground and sieved through a 200–250 μm sieve.	pH of 1 Contact time: 1 h Initial dye concentration: 900 mg/L	Maximum adsorption capacity: 324.88 mg/g	Langmuir isotherm model	Pistachio husk powder turned out to be highly efficient in adsorption of reactive red 120, dependent on initial dye concentration, pH level, and contact time.		[197]
Tetracycline (TEC)	Pistachio shell (coated with ZnO nanoparticles)	Washed with distilled water, soaked, and dried at 105 °C. Dried shells were milled and sieved to different particle size ranges: 194, 122, and 87 μm; some amounts were stored for ZnO nanoparticles coating.	pH of 5; particle size of 87 μm; the CPS adsorbent dose of 0.08 g/100 mL. Shaking speed for adsorption: 150 rpm. Initial TEC concentration: 70 ppm	Adsorption of TEC on the nanoparticle-supported adsorbents; 95 mg/g. Removal efficiency at optimum conditions: 84.87%	Freundlich Isotherm model	CPS can adsorb TEC, AMO, and CIP in alkaline, neutral, and acidic environments. A higher speed can reduce the removal of contaminants and not allow sufficient time for adsorption due to the heterogeneity of the adsorption mixture (vortex phenomenon).	Both studies show that pistachio shells can be effectively adsorbed on ZnO nanoparticles for purification of wastewater contamined with antibiotics; they may hence be used for cleaning post-hospital waste.	[198,199]
Tetracycline (TEC), amoxicillin (AMO), and ciprofloxacin (CIP)	Pistachio shell (coated with ZnO nanoparticles)	Washed with deionized water, dried at 105 °C, grounded, and sieved. Coated with ZnO nanoparticles.	pH of 5; 30 min of contact time for TEC and CIP and 60 min for AMO.	Highest adsorption capacity AMO (132.240 mg/g) TEC (98.717 mg/g) CIP (92.450 mg/g)	Freundlich Isotherm model			
Sarafloxacin	Raw pistachio nutshells (RPNSs)	-	pH 5.0–6.0	Removal efficiency Raw pistachio nutshells: 82.39% Multi-walled carbon nanotubes: 96.20%	Elovich isotherm model	Raw pistachio shells turned out to be efficient for adsorption and removal of the veterinary fluoroquinolone antibiotic sarafloxacin (SARA). It may be used in bioremediation of wastewater from anti-Gram(−) and anti-Gram(+) antibiotics.		[181]
Brilliant green	*Pistacia vera* shells	*Pistacia vera* shells were ground and mercerised with 5% NaOH to enhance surface properties.	Initial dye concentration of 10.1 mg/L and sorbent dose of 4.0 g/L.	Maximum removal efficiency of 98.78%; maximum adsorption capacity decreasing with temperature; 52.42 mg/g at 300 K.	Sips isotherm model	The NaOH treatment removed impurities and improved surface accessibility, resulting in ground shells becoming useful for the adsorption of brilliant green cationic dye from wastewater.		[200]
4-nitrophenol (4-NP), methylene blue (MB), rhodamine B (RhB), methyl orange (MO)	Pistachio hull extract with copper nanoparticles (CuNPs)	Fruits were peeled, washed with distilled water, dried at 60 °C, pulverised, and sieved; suspended in distilled water, cooled, and separated from plant tissues; the aqueous extract was filtered and separated. Used in biosynthesis of CuNPs and Cu/PS NC.	Ambient temperature; in form of copper nanoparticles; waste pistachio shell covered in nanoparticles; copper/pistachio nanocomposite.	Cu/PS NC86 4-NP: more than 99% in reduction CuNPs 4-NP: about 97%	-	The biosynthesized CuNPs and copper/pistachio shell nanocomposite (Cu/PS NC) efficiently reduced 4-nitrophenol, methylene blue, rhodamine B, and methyl orange at ambient temperature. The composite was found to be reusable and recyclable, without a decrease in catalytic activity.		[201]
Crystal violet	*Pistacia lentiscus* leaf powder	Washed with demineralised water, air-dried, ground, and sieved	60 min; pH of 7.1; 30 mg biosorbent (80 μm particle size).	Maximum removal of 98.25% and maximum adsorption capacity of 93.03 mg/g	Freundlich isotherm model	Pistachio leaf powder enabled environmentally friendly and cost-effective removal of the crystal violet dye from textile waste.		[202]
Remazol red (azo dye)	Pistachio internal shells	The pistachio shells were crushed, grounded, and sieved to obtain particle size in the range of 250–2000 μm; dried at 110 °C	pH of 2; 10 min of mixing time; initial dye concentration at 150 mg/L; temperature of 20 °C.	Highest adsorption capacity: 108.15 mg/g at 20 °C	Freundlich isotherm model	This study suggests that pistachio shells can potentially be used as a low-cost adsorbent for eliminating azo dyes as high removal of Remazol red has been proven.		[203]
Malachite green	*Pistacia vera* shell-based active carbon	Washed under tap water and soaked in double-distilled water for 5 h; pyrolysed, pulverised, and sieved with a 240-μm sieve mesh.	pH of 7; initial dye concentration of 75 mg/L.	99.9% removal of malachite green; 76.92 mg/g maximum adsorption capacity	Langmuir isotherm model	Tested active carbon effectively removed the cationic dye pollutant and the material was found to be reusable for four consecutive cycles.		[24]
Methylene blue	*Pistacia vera* shell	Washed, dried, treated with hydrogen peroxide (30% *w*/*w*), and kept in a water bath at 50 °C for 60 min with constant stirring at 100 rpm. Afterwards the biomass was treated with a 0.10 M solution of NaOH, washed, and dried.	pH of 5.63; ionic salt concentration as low as possible.	92.12% biosorption in optimal conditions	Sips isotherm model	The treatment significantly improved the biosorption properties and the material could be recovered with 99.8% efficiency using 0.15 M oxalic acid, with regeneration studies showing very good recovery rates for four consecutive cycles.		[204]
Methylene blue	*Pistacia* sp. hull waste	-	70 min contact time, pH of 8, pistachio hull powder concentration of 1.5 g/L; higher temperature and lower dye concentration improved adsorption.	99.7% removal efficiency; maximum adsorption efficiency of 602 mg/g at 50 °C	Langmuir isotherm model	Pistachio powder exhibited good adsorption properties in the treatment of methyl blue-contaminated wastewater, while remaining inexpensive and environmentally friendly.		[205]
Methylene blue	*Pistacia khinjuk* Stocks leaf extract	Leaves were washed, shade-dried (96 h), and ground. Ultrasonic-assisted extraction (45 °C, 45 min, 37 kHz), mixed with zinc acetate solution (10 mM), stirred 1 h at 45 °C; centrifuged, washed, dried (250 °C, 2 h), and collected as nanoparticles.	Dye concentration: 50 ppm Catalyst dose: 10–15 mg (optimal: 15 mg) UV irradiation time: 150 min Pre-equilibrium (dark): 25 min Light source: UV	Max degradation: 97.6% (15 mg ZnO)	Not applicable	The study demonstrates high photocatalytic potential of green synthetized ZnO nanoparticles, presenting Pistacia leaf extract as efficient green nanosynthesis component.		[206]
Methylene blue	*Pistacia* sp. green outer shell	*Pistacia* sp. green outer shells were used for the preparation of hydrochar in a stainless steel pressurised reactor in a subcritical water medium.	Temperature of 318 K; activation with 1 M KOH solution.	Maximum adsorption capacity of 17.92 mg/g at 318 K	Langmuir isotherm model	Hydrochar effectively adsorbed the dye, with the process being spontaneous and endothermic.		[207]

**Table 7:** Potential forms of application of *Pistacia* sp. in the adsorptive removal of heavy metals and elements.

Pollutant	Form and Species of *Pistacia*	Method of Preparation	Optimal Experimental Conditions		Efficiency and Adsorption Capacity	Adsorption Model	Note	Reference
Heavy metals (Pb and Cu)	Roasted and lightly salted *Pistacia* sp. shells	Soaked in warm water, oven-dried; pyrolyzed at temperature varying between 250 and 650 °C in the oven under nitrogen.	10 g/L of biochar produced after pyrolysis at 550 °Cin the time scope of 1 h.		Pb and Cu adsorption at 99.7% and 99.6% respectively at the initial concentration of 15 mg/L.	Freundlich	Pyrolysis of pistachio shells produced biochar suitable for heavy metal adsorption from wastewater. Pyrolysis temperature increases Pb and Cu adsorption capacity.	[208]
Cadmium [Cd(II)], Lead [Pb(II)]	*Pistacia vera* hull	Washed with deionized water; dried at 50 °C for 24 h, ground, and sieved through a 270-mesh sieve.	Cd(II): 30 min Pb(II): 120 min	Cd^2+^ pH range of 7.0–8.0 Pb^2+^: pH lower than 8	Biosorption capacity Cd(II): 87 and 90% at 5 and 48 mg/L respectively Pb(II): 90% at both 88.7 mg/L and 883 mg/L	Sips	Biosorption of Cd(II) and Pb(II) is pH-dependent showing a maximum value at pH 8.0. *Pistacia vera* hulls from aqueous solution demonstrated great biosorptiove activity with no prior chemical modification.	[209]
Calcium, magnesium	*Pistacia vera* shell	Dried at 60 °C and blended.	pH of 8		Maximum adsorption capacity Ca: 2.41 mg/g and 21.84% at 1.5 g of *P. vera* shell Mg: 2.19 mg/g and 14.86% at 2.5 g of *P. vera* shell	Ca: Langmuir Mg: Freundlich	The study shows that, potentially, the tested shell can be used for softening hard water in a biodegradable and inexpensive way.	[210]
Chromium(VI)	*Pistacia* sp. green hull	Air-dried for 3 days, crushed, powdered, and passed through a 200-mesh sieve.	Temperature of 40 °C, pH of 2; optimal time and pistachio hull powder concentration depend on the chromium(VI) concentration.		Maximum adsorption capacity of 116.3 mg Cr(VI) per g of pistachio hull powder.	Langmuir for equilibrium and pseudo-second order for kinetic characteristics	Pistachio hull powder can be used for effective removal of Cr(VI) from wastewater, while remaining simple and cost-effective.	[211]
Chromium(VI)	*Pistacia atlantica* leaf extract	*Pistacia atlantica* leaf extract was used as a reducing agent in the synthesis of iron nanoparticles.	pH of 2; Cr(VI) concentration of 25 mg/L; adsorbent dose of 24 mg/L.		99.9% in proper conditions; maximum adsorption capacity of 2.585 mg/g.	Langmuir	*Pistacia atlantica* leaf extract is suitable for the synthesis of adsorbent materials, offering effective removal and eco-friendliness at a low cost.	[212]
Cu(II)	*Pistacia* sp. shell	Pistachio shells were washed, dried, ground, and sieved to an appropriate particle size using a 44–52 mesh.	pH of 5.9		Adsorption capacity of 33.25 mg/g.	Langmuir	Pistachio shells were proven to be suitable for Cu(II) removal from wastewater. It is possible to effectively regenerate the treated shells with 0.4 N HCl and use the adsorbent in the presence of other metals.	[213]
Nickel (II)	*Pistacia* sp. hull waste (PHP)	Pistachio hull waste was dried, crushed, sieved (mesh 14–100), and washed, before being dried again.	pH between 4 and 6; PHP dosage of 25 g/L; no notable changes in nickel removal after 40 min.		Maximum adsorption capacity of 14 mg/g.	Freundlich	Powdered pistachio hull effectively removed nickel ions, with optimal conditions allowing for fast and efficient adsorption.	[214]
Uranium (VI)	*Pistacia vera* shell	*Pistacia vera* shells were used for the preparation of activated carbon through physicochemical activation using CO_2_ at 700 °C for 2 h.	pH of 3; adsorption equilibrium reached at 90 min; the process was found endothermic, with rising temperature increasing removal up to the highest tested at 318.15 K.		Maximum monolayer adsorption capacity of 8.68 mg/g.	Langmuir	Activated carbon prepared from *Pistacia vera* shell was proven effective in the removal of uranium.	[215]
Uranium	*Pistacia* sp. shell	*Pistacia* sp. shells were washed with a buffer and added to a buffer solution containing uranyl nitrate.	pH of 4; equilibrium achieved after 2 h contact time; 15 °C or higher.		Uranyl adsorption capacity in seawater at 70 μg/g; at the concentration of uranyl ions of 100 ppm, experimentally determined adsorption capacity was 355 μg/g.	Freundlich	Pistachio shells were found to be suitable for the removal of uranyl-contaminated sea water.	[216]
Heavy metals (Pb, Cu, Co, Ni)	*Pistacia vera* hull	Pistachio hull waste was washed, dried, ground, and sieved through a 200-mesh sieve.	Highest performance at pH 11; used pH 7 for practical reasons, as well as little increase with the change from pH 7 to 9. Optimal adsorbent dosage was found to be 45 mg; contact time 90 min.		Adsorption efficiencies of 87%, 73%, 69%, and 88% for Pb, Cu, Co, and Ni respectively in 60 min for 45 mg of pistachio hull waste.	Freundlich	The pistachio waste achieved high levels of heavy metal adsorption, showing its potential for eco-friendly water treatment.	[217]
Mainly Cu(II); Fe(II), Zn(II), Ni(II)	*Pistacia* sp. shell	Magnetic activated carbon nanocomposites modified by sulfamic acid were developed with pistachio shell as a precursor.	Contact time of 180 min; Cu(II) concentration of 60 mg/L; nanocomposite dosage of 0.6 g/L; initial pH of 6.5.		Maximum adsorption capacity of Cu(II) at 277.77 mg/g; 95.29% removal at optimal conditions	Langmuir	The created nanocomposite was found to be an efficient bioadsorbent for heavy metals, with good reusability at up to seven cycles with high performance.	[218]
11 heavy metals including Cr, Mo, and Sb	*Pistacia lentiscus* leaf extract	*Pistacia lentiscus* leaf extract was used in the synthesis of MgO@SnO_2_ nanocomposites; surface modified with polyvinylpyrrolidone.	30 min contact time; sunlight exposure for hydrocarbon photocatalytic degradation.		Complete Cr, Mo, and Sb removal after 20 min; 99% removal of all tested metals after 30 min.	Freundlich	The synthesised nanocomposite showed high suitability for heavy metal removal, while also having potential for photocatalytic degradation of hydrocarbons.	[219]
Lead	*Pistacia* sp. shell	Pistachio shell was used for the preparation of biochar via pyrolysis at 300, 450, and 600 °C.	pH of 6.		2.5 mg/g; 20–35% Pb^2+^ removal.	Langmuir	While pistachio biochar was not found to be the most efficient of the tested materials, it still demonstrated some capacity for heavy metal adsorption.	[220]
Mercury (II) (Hg^2+^)	*Pistacia* sp. green shell	Pistachio green shells were washed, dried, ground, and sieved with 100-μm mesh, before being blended with activated carbon.	pH of 6.13; pistachio shell powder dosage of 9.21 g/L; initial mercury (II) concentration of 36.68 g/L; activated carbon dosage of 7.25 g/L.		99.25% Hg removal.	Langmuir	Pistachio shells mixed with activated carbon easily removed Hg^2+^ from wastewater.	[221]
Zinc (II)	*Pistacia vera* seed	*Pistacia vera* shells were washed, dried, ground, and sieved to a particle size of 0.5 mm.	pH of 6; contact time of 10 min; adsorbent dose of 8 g/L.		Under optimal conditions the removal rate was found to be 96.7%.	Pseudo-second order for kinetic characteristics	Pistachio shells formed part of an effective adsorbent for zinc (II) ion.	[222]
Lead (II) (Pb^2+^)	*Pistacia* sp. hard shell	*Pistacia* sp. hard shells were used for the preparation of composite matrix of shell-grafted-thiosemicarbazone acetophenone.	pH of 5; contact time of 2 h, modified adsorbent dosage of 0.165 g; removal rates increasing with temperature (endothermic process).		Maximum removal rate of 97.08%.	Freundlich for equilibrium and pseudo-second order for kinetic characteristics	The synthesised composite demonstrated high capacity for lead adsorption, making it feasible for treatment applications.	[223]
Copper (II)	*Pistacia* sp. green hull	*Pistacia* sp. green hulls were washed, dried, and pyrolysed at 450 °C for 1 h in the absence of oxygen.	pH of 5; solid–liquid ratio of 0.3 g/L.		Maximum sorption capacity calculated via the Langmuir model was 19.84 mg/g.	Langmuir	The obtained biochar was found to be a cost-effective and eco-friendly adsorbent that can be used to remove copper (II) from wastewater.	[224]

**Table 8:** Potential utilisation of *Pistacia* sp. in the development of biofuels.

*Pistacia* Species	Part of Plant/Material	Type of Fuel	Obtaining Method	Results	Reference
*Pistacia chinensis* Bunge	Seed oil	Biodiesel	Amino-modified composite graphene oxide/cellulose microspheres were prepared and used as catalyst in the production of biodiesel from *P. chinensis* seed oil.	The created biodiesel was found to meet or exceed all EN 14214 standards for biodiesel quality. The catalyst used enabled a transesterification reaction with a 94% yield of fatty acid methyl esters (FAMEs)	[229]
*Pistacia vera* L.	Soft shell	Bio-oil	Pyrolysis was performed on *P. vera* soft shells in a flat-bed reactor to produce bio-oil. Different conditions were tested for their effects on the final product.	Under optimal conditions, the maximum oil yield was found to be 33.18%, with the product having an H/C molar ratio of 1.43 and a gross heating value of 33.78 MJ/kg; i.e., comparable to petroleum fractions.	[230]
*Pistacia vera* L.	Dehulling waste (epicarp, peduncles, leaves, mesocarp, kernel to a lesser extent)	Biogas	*P. vera* waste from dehulling (wastewater, solid waste, and their mixtures) were subjected to anaerobic digestion to produce biogas, including methane.	The process showed potential in methane production, with solid waste outperforming some high-solid materials such as animal manure and food processing residues.	[231]
*Pistacia vera* L.	Dehulling waste (epicarp, peduncles, leaves, mesocarp, kernel to a lesser extent)	Biogas	Biogas was produced from pistachio dehulling waste by anaerobic bacteria. Biogas yield, anaerobic treatability, and the effect of different pretreatments on the process were tested.	Methane production using pistachio dehulling waste was proven possible, with proper pretreatments allowing for methane yield as high as 213.4 mL CH_4_/g COD.	[232]
*Pistacia vera* L.	Shell	Bioethanol	Ozone and hot water pretreatments were applied to pistachio shells to prepare them for the production of bioethanol via enzymatic hydrolysis.	Fermentation efficiency was found to be between 42% and 55%, showing that pistachio shells can be used for bioethanol production, given proper pretreatments.	[233]
*Pistacia lentiscus* L.	Seed oil	Biodiesel	A single-step homogenous alkali-catalysed transesterification process was used on *P. lentiscus* seed oil to produce biodiesel.	The synthesised biodiesel demonstrated 3% higher thermal efficiency than standard diesel fuel, in addition to lower carbon dioxide, unburned hydrocarbon, and particulate matter emissions. However, higher brake specific fuel consumption and nitrogen oxide (NO_x_) emissions were recorded.	[234]
*Pistacia* sp.	Shell	Gas	Pistachio shells were used to produce fuel gas using a bubbling fluidised bed gasifier under air and steam atmospheres.	The treatment yielded fuel gas with a lower heating value: 3.2–5.4 MJ/Nm^3^ under air gasification (equivalence ratio 0.2–0.4) and 9.6–9.9 MJ/Nm^3^ under steam gasification.	[235]
*Pistacia khinjuk* Stocks	Seed oil	Biodiesel	Using esterification and transesterification, oil from *P. khinjuk* was used to produce biodiesel. The obtained fuel was mixed with different proportions of conventional diesel to test their performance together.	Mixing the synthesised biodiesel with diesel reduced carbon monoxide, smoke opacity, and unburned hydrocarbon, while increasing the nitrogen oxide (NO_x_) emissions and fuel consumption.	[25]
*Pistacia terebinthus* L.	Waste oil	Biodiesel	Calcium and magnesium tablet pharmaceutical waste was calcinated and used as a catalyst for biodiesel production from *P. terebinthus* using transesterification.	The maximum mass efficiency of 96% and qualitative efficiency of 91.37% was achieved in optimal conditions, with the former decreasing to 71.4% after four cycles of the catalyst. Compared to conventional diesel, biofuel had lower external costs.	[236]
*Pistacia* sp.	Tree pruning residues	Pellet	Pellets were made from pistachio tree pruning residues by pressing ground material of different moisture content levels and particle size and at different pressing temperatures.	It was found that the pellets exhibited the best density, strength, and durability, as well as lowest friction at 11.7% moisture content, 100 °C pressing temperature, and 1.65 mm particle size.	[168]

**Table 9:** Potential insecticide, acaricide, repellent, and fumigant activity of extracts and oils from *Pistacia* sp.

Form of *Pistacia*	Target Species	Extract Type and Preparation Method	Dose/ Concentration	Main Active Ingredients/Collected Compounds	Efficacy (e.g., LC_50_)	Mode of Action	Effects and Methods	Reference
Unripe fruits of *Pistacia therebinthus*	*Acanthoscelides obtectus*	Essential oil obtained through water steam distillation using a Clevenger apparatus; diffused using dental cotton.	-	-	LC_50_ = 10.2 µL/L for males LC_50_ = 14.9 µL/L for females	Fumigant toxicity	Unclear, although potential repellent effect—the essential oil caused minor adverse effect on adult emergence.	[241]
Leaves of *Pistacia lentiscus* L.	*Aphis spiraecola*, *Aphis* *gossypii*	Essential oil obtained through hydrodistillation using modified Clevenger-type apparatus.	400, 500, 600, 800, 900, and 1100 ppm	α-pinene, β-myrcene, L-limonene, camphene, sabinene, bornyl acetate trans-caryophyllene, cymene, 4-terpineol	*A. spiraecola*: LC_50_ = 759 ppm *A. gossypii*: LC_50_ = 490 ppm	Contact insecticidal activity	There was no significant difference in toxicity of the tested essential oil and the chemical insecticide used as positive control, suggesting that it can be a green substitute for chemical control of targeted insect species. The essential oil has potential as a biopesticide in integrated pest management.	[242,243]
	*Myzus persicae*				*M. persicae*: LC_50_ = 596 ppm LC_95_ = 1264 ppm			
Twigs of the Seedlings of *Pistacia chinensis*	*Batocera* *horsfieldi*	Isolated volatile compounds obtained through dynamic enclosure technique; diluted in hexane + n-dodecane.	1 mg/mL, 10 mg/mL, 100 mg/mL	α-pinene, β-pinene, α-phellandrene, D-limonene, β-ocimene, (Z)-3-hexen-1-ol, 3-carene, γ-terpinene	The selection rate for D-limonene: 11.11% for females and 11.30% for males repelled	Olfactory repellent	All tested compounds caused EAG responses. However, only D-limonene, a volatile compound present in the tested plant, has an obvious repellent effect on *B. horsfieldi.*	[244]
*Pistacia atlantica* subsp. *kurdica* (gum, fruit, and leaves) and Pistacia khinjuk (fruit and leaf)	*Callosobruchus maculatus*	Essential oils obtained through water steam distillation using a Clevenger apparatus.	-	Spathulenol, camphene, β-myrcene, β-pinene, D-limonene, β-ocimene, E-nerolidol	*P. atlantica* subsp. Kurdica Gum LC_50_ = 7 µL/L Fruit LC_50_ = 18 µL/L Leaves LC_50_ = 24 µL/L *P. khinjuk* fruit: LC_50_ = 22 µL/L Leaves: LC_50_ = 24 µL/L	Fumigant toxicity	*Pistacia* sp. essential oils showed significant repellent activity in all concentrations *C. maculatus* mainly after two and four hours of exposure. At the highest concentration of 0.0234 µL/cm^3^, for *P. atlantica* subsp. Kurdica, the respective repellency percentages for gum leaves and fruit oils were 82%, 77%, and 70% after two hours, and for *P. khinjuk*, the respective repellency percentages for leaves and fruit oils were 76% and 67%.	[245]
Leaves of *Pistacia lentiscus*	*Dermanyssus gallinae*	Essential oil obtained by hydrodistillation for 3 h using Clevenger apparatus.	0.43, 0.87, 1.75, and 3.5 mg/cm^2^	α-pinene (20.58%), D-limonene (18.16%), β-Myrcene (15.06%), 4- T terpineol (7.68%), caryophyllene (5.45%), and γ-terpinene (5.21%)	Spraying LC_50_ = 0.354 mg/cm^2^ Contact LC_50_ = 2.561 mg/cm^2^	Acaricide effect; contact toxicity; spraying toxicity	The tested essential oil and its active components individually exhibited a substantial acaricidal activity on *D. gallinae*. Spraying was more effective than contact treatment.	[246]
Leaves of *Pistacia lentiscus*	*Ectomyelois ceratoniae* Zeller and *Ephestia kuehniella* Zeller	Essential oil obtained after subjecting leaves to hydrodistillation using a modified Clevenger-type apparatus.	Between 20 and 160 mL/L	Terpinene-4-ol (23.32%), α-terpineol (7.12%), and β-caryophyllene (22.62%)	*E. kuehniella* LC_50_ = 1.84 mL/L, LC_95_ = 5.14 mL/L *E. ceratoniae* LC_50_ = 3.29 mL/L LC_95_ = 14.24 mL/L	Fumigant toxicity; lowering hatching rate	Fumigant toxicity test proved that the tested oil exerted higher toxicity against *E. kuehniella* than *E. ceratoniae.* Higher concentrations and exposure times were associated with lower fecundity and hatching rates for both insects. Copulation rate among tested insects significantly decreased after exposure to essential oil.	[247]
*Pistacia lentiscus* fruit, leaves, and bark	*Lobesia botrana*	Crude hydromethanolic extracts; prepared through maceration in methanol and homogenization.	10, 20, 40, 80, 150, 160, 200 µg	Fatty acids: oleic and linoleic acid	Fruit extract: After 3 h LC_50_ = 441.2 µL/cm^3^ After 24 h LC_50_ = 287.85 µL/cm^3^	Insecticidal and larvicidal effect—topical application	Extracts obtained from leaves and bark exerted no significant larvicidal effect. Metabolites and components of fruit extract were toxic on tested larvae, particularly oleic and linoleic acid.	[248]
*Pistacia lentiscus*	*Sitophilus granarius*	Essential oils	0, 25, 50, and 100 µL/kg	Monoterpene hydrocarbons Oxygenated monoterpenes Sesquiterpene hydrocarbons Oxygenated sesquiterpenes Diterpene hydrocarbons Phenylpropanoids Non-terpene derivatives	LC_50_ = 36.36 µL/kg	Olfactory profile and toxicity	Co-treatment with *P. lentiscus* essential oils and diatomaceous earth enhanced the insecticide efficacy of the two substances. Combined treatment also reduced the olfactory characteristics of the grain.	[249]
Leaves of *Pistacia lentiscus*	*Tribolium castaneum* and *Lasioderma* *serricorne*	Essential oil obtained through hydrodistillation using a modified Clevenger-type apparatus.	5 and 45 μL corresponding respectively to concentrations of 114 and 1023 μL/L air.	α-phellandrene (3.20%), α-pinene (9.48%), and limonene (19.11%)	*T. castaneum* LC_50_ = 28.03 μL/L LC_95_ = 63.46 μL/L *L. serricorne* LC_50_ = 8.44 μL/L LC_95_ = 43.68 μL/L	Fumigant toxicity	The tested essential oil may have potential as a control agent against two beetles known to attack stored products: *L. serricorne* and *T. castaneum*. Greater toxicity was observed for *L. serricorne*.	[250]
Gum, fruit, and leaves of *Pistacia atlantica* subsp. *kurdica*	*Tribolium castaneum*		14–71 µL/L air for gum oil, 41–55 µL/L air for fruit oil, and 57–71 µL/L air for leaves oils.	Gum: α-pinene, terpinolene, β-pinene, camphene Fruit: α-pinene (47.7%), β-myrcene (16.1%), D-limonene (8.75%) Leaves: spathulenol (24.1%), α-pinene (19.2%), and δelemene (7.05%)	Gum: LC_50_ = 29 µL/L air Fruit: LC_50_ = 39 µL/L air Leaves: LC_50_ = 64 µL/L air	Fumigant toxicity	The strongest insecticidal activity and toxicity was exerted by gum essential oil in comparison to fruit and leaf-derived oils. The activity was determined by calculation of mortality rate and antenna movement attenuation.	[27]
Essential oils from *Pistacia atlantica* Desf. and *Pistacia lentiscus* L.	*Tribolium confusum* Dul.	Essential oils; the aerial parts of the plants were hydrodistilled in a Clevenger-type apparatus.	5, 10, 15, and 20 µL/L air	*P. lentiscus*: (E)-β-caryophyllene (16.3%) and γ-cadinene (15.6%) *P. atlantica*: terpinen-4-ol (35.6%)	*P. atlantica* LC_50_ = 15 ± 1.1 µL/L air *P. lentiscus* LC_50_ = 7.5 ± 0.8 µL/L air	Fumigant toxicity Corrected mortality of essential oils	The essential oils exhibited strong fumigant toxicity against the tested insects, with multiple oils demonstrating synergistic action. *P. lentiscus* extract achieved a higher mortality rate among the tested insects than *P. atlantica.*	[251]
Green husk methanolic extract of *Pistacia khinjuk*	*Tribolium confusum* Dul. and *Oryzaephilus surnamensis*		2, 4, 8, 20, 30, 50 mg/mL	-	*T. confusum* Dul. LC_50_ = 9.32 mg/mL air *O. surnamensis* LC_50_ = 5.47 mg/mL air	Contact toxicity	Mortality of *O. surinamensis* observed with 8 mg/mL: 62.5% Mortality of *T. confusum* observed with 8 mg/mL: 55%	[252]
*Pistacia atlantica* Desf.	*Ixodes ricinus* L.	Essential oil produced through steam distillation and n-pentane extraction	20% in acetone	Monoterpene hydrocarbons, sesquiterpenes (germacrene D, bourbonene), and alcohols (terpinene-4-ol)	-	Scent attraction and repellence	The essential oil showed a significant repellence after 20 min to one hour. The tested oil may be a good but short-lasting tick repellent.	[253]

**Table 11:** Application of *Pistacia* sp. in Available Patents.

Category	Patent Number	Title	Technology Description and Application
Agriculture	US11739031B2	Biochar encased in a biodegradable material	Pistachio shell biodegradable polymer-coated biochar particles used for seed coating and soil amendments. Enhances moisture control, microbial growth, and nutrient delivery. Designed for sustainable agriculture.
	US10959384B2	Plant substrate growing medium	A plant growth substrate using nutshells—including *Pistacia* (pistachio) shells—composted, buffered, and potentially mixed with peat or coir to create a soilless medium. Especially useful for small fruit cultivation by providing porosity, moisture control, and structural support.
Food preservation	WO2016111659A1	Edible antimicrobial film made of pistachio resin	An edible film was prepared using pistachio resin dissolved with vital gluten in ethyl alcohol, plasticized with glycerol or glutaraldehyde, centrifuged, and dried. The resulting flexible and edible sheet can be potentially used in food preservation with antimicrobial properties inherent from pistachio resin. The material is natural, biodegradable, and safe for direct contact or ingestion.
Insecticidal and repellent activity	CN106342942A	Plant-sourced mosquito repellent incense and preparation method thereof	Constituents present in raw material from *Pistacia weimannifolia* in synergy with other components led to invention of an efficient mosquito repellent incense, with its special advantages being environment-friendly, healthy, simple, and convenient in use method, as well as low in cost, with inhibiting and killing effect to viruses and bacteria.
	CN104094977B	Composition with insect-expelling sterilisation disinfection effects and application thereof	The described invention relates to a mixture based on crude vegetal used to expel parasites, sterilise, and disinfect. It appears to be an environmentally friendly, nontoxic, harmless, and natural parasite-expelling, sterilisation, and disinfection agent.
Fuels	US10538433B2	Activated carbon production at biomass-fuelled steam/electric power plants	*Pistacia* wood is cited as an example of biomass feedstock for activated carbon production at biomass-fuelled steam/electric power plants. Activated carbon can be used onsite (e.g., pollution control) or sold, improving energy efficiency, reducing waste, and enabling carbon credit opportunities.
	US10961459B2	System for production of a renewable liquid fuel	The invention includes a method for compounding a non-aqueous biofuel derived from various solid, processed biomass furnishes into a liquid fuel that may be used in internal combustion engines such as diesel engines. Pistachio shells represent a practical, commercially viable, and functional source of low-cost biomass.
Purification and adsorption	CN106944001B	Preparation method of biological carbon adsorbent	The process of preparing a carbon adsorbent using pistachio shell powder; the resulting biochar demonstrates high adsorption and can be used to create a bio-carbon/tourmaline adsorbent after mixing with pretreated tourmaline. The adsorbent was revealed to be efficient in removal of chromium, lead and methylene blue
	CN106732368A	A kind of hazelnut shell, *Fructus Pistaciae Verae* shell/tourmaline adsorbent, and preparation method thereof	
	CN106732369A	A kind of pinenut shell, *Fructus Pistaciae Verae* shell/limonite adsorbent, and preparation method thereof	Production of a high-performance pistachio nutshell biochar preparation that can be used to prepare a bio-carbon/limonite adsorbent. After proper processing, biochar exerts high adsorptive potential associated with the removal of chromium, lead, and methylene blue.
	CN106732370A	A kind of hazelnut shell, *Fructus Pistaciae Verae* shell/limonite adsorbent, and preparation method thereof	
Nanotechnology use	KR101865712B1	Method for production of mastic gum solution with high dispersion activity and solubilization activity by nanoparticle system	The invention describes the process of forming nano-sized particles using milling and stabilising agents (gum arabic, xanthan gum, cyclodextrin, glycerin, or biogums) in controlled temperature to enhance dispersibility and solubility in water. High aqueous solubility allows for the use of *Pistacia* mastic gum in cosmetics, food, beverages, dental products, etc., with pharmaceutical and clinical use.
Cancer treatment	US8722109B1	Composition comprising plant extracts and essential oils	Mastic resin from *Pistacia* sp. was used in preparation of a natural solid formulation administered with essential oils as a bioactive agent with therapeutic benefits. The invention is intended as a natural tumour treatment in alternative application and in mitigation of side effects of chemotherapy and radiation.
Materials and packaging	US20130130963A1	Packaging for a liquid detergent with abrasive particles	Ground pistachio nutshells can be used as natural abrasive particles in liquid detergent packaging. Creating packaging with abrasion resistance improves dispensing of liquids.
	US 12121944B2	Three-dimensional printed compositions using organic substrates such as coffee, pistachio shells, and coconut hells, with bacteria-based binders, coatings for three-dimensional printed compositions, and processes related to the same	Pistachio shells used in providing 3D-printing materials combined with enzyme-producing bacteria that induce microbial calcite precipitation to bind the printed material; as a form of eco-friendly and biodegradable material, it may be a great option for zero-waste production of everyday amenities. The material can be metallized for an aesthetic finish.
	JP7498968B2	Flash Joule heating synthesis method and compositions thereof	Rapid flash Joule heating process used in conversion of carbon into turbostatic graphene; pistachio shells pose as a carbon source for obtaining graphene used in composites to boost strength, conductivity, and performance.
	WO2022109723 A1	Polyurethane elastomer compositions, articles of manufacture comprising the same, and processes	Bio-based polyurethane elastomer formulation invention with the use of pistachio nutshell as a bio-additive for hardness tailoring, toughness, and reduction of reliance on synthetic plasticizers or polyols.
Cosmetics	US20120082737A1	Topical skin care formulations comprising plant extracts	Topical application in a composition of multiple extracts; administered in various forms (emulsions, anhydrous bases, gels, ointments, etc.) in dermal care, reducing the effects of environmental impact and ageing on skin features, erythema, dehydration, etc.
	KR101865712B1	Method for production of mastic gum solution with high dispersion activity and solubilization activity by nanoparticle system	Achievement of nano-dispersion of *Pistacia lentiscus* “mastic” gum with an increase in water solubility, allowing for the use of mastic gum in cosmetics.
	WO2019170239A1	Terpene-enriched fractions free from polyterpenes extracted from Chios mastic gum and cosmetic, nutraceutical, medical devices, and pharmaceutical compositions containing them	Extraction and enrichment of terpene fractions from *Pistacia lentiscus* “mastic” gum resulting in achievement of high purity monoterpenes suitable for cosmetic formulations targeted in skin repair and fighting inflammatory responses.
	WO2010030082A3	Compositions comprising acidic extracts of mastic gum	Synergistic effect of constituents of the acidic fraction of *Pistacia lentiscus* “mastic” can be useful in treating conditions associated with impaired neuronal functions, promoting wound healing and rejuvenating cells and tissues.
	JP7227903B2	Method for producing mastic gum extract and mastic gum extract	The invention provides a method for producing *Pistacia lentiscus* “mastic” gum extract involving processing to obtain purified extract with various bioactive compounds (terpenes, phenolic compounds, etc.). Preservation of these compounds leads to potential incorporation of obtained extract in oral hygiene, skin care, or even peptic ulcer treatment through anti-inflammatory, antioxidant, and antimicrobial properties.

## Data Availability

No data was used for the research described in the article. Data sharing is not applicable.
